# Abietane Diterpenes of the Genus *Plectranthus sensu lato*

**DOI:** 10.3390/molecules27010166

**Published:** 2021-12-28

**Authors:** Mária Gáborová, Karel Šmejkal, Renata Kubínová

**Affiliations:** Department of Natural Drugs, Faculty of Pharmacy, Masaryk University, 612 00 Brno, Czech Republic; smejkalk@pharm.muni.cz (K.Š.); kubinovar@pharm.muni.cz (R.K.)

**Keywords:** abietane, coleon, diterpene, hydroquinone, lanugon, *Plectranthus*, royleanone, spirocoleon

## Abstract

*Plectranthus* (Lamiaceae), which—according to the latest systematic revision—includes three separate genera (*Coleus*, *Plectranthus sensu stricto*, and *Equilabium*), is a genus widely used in traditional medicine—mainly in the treatment of various ailments of the digestive tract, respiratory tract, and skin. Many species of *Plectranthus s.l.* have been shown to produce phenolic compounds and terpenes. Diterpenes, especially those of the abietane class, are the most studied group of secondary metabolites found in *Plectranthus s.l.*, which is characterized by a significant structural diversity arising from the oxygenation and further rearrangement of the basic tricyclic abietane skeleton to a complete aromatization of the ring system. This review summarizes the known information on abietane diterpenes, showing their structures, sources, and biosynthesis. A classification of these compounds into nine groups, according to the arrangement of their ring C, is used. Royleanones, spirocoleons, and hydroquinones are the largest classes of abietane diterpenes, covering more than 70% of all the compounds reviewed.

## 1. Introduction

Since early human history, medicine has been closely linked with natural products—especially with plants, which have played a key role in treating various diseases for centuries. The fact that approximately 25% of conventional medicines prescribed worldwide are of plant origin proves that there is still room for further investigation in the field of phytochemistry, leading to the discovery of compounds with remarkable biological activity. Such natural compounds may be used directly in treatments or as initial steps in drug discovery, resulting in the synthesis of analogues with improved pharmacokinetic and pharmacodynamic profiles [1].

The genus *Plectranthus sensu lato* (Lamiaceae, subfamily Nepetoideae) comprises three genera: *Coleus* (294 species), *Plectranthus sensu stricto* (72 species), and *Equilabium* (42 species) [2,3], and includes soft trailing semi-succulent to succulent herbs and shrubs, which are widely distributed in the summer-rainfall savannahs and forested regions of Africa, Madagascar, India, Australia, and a few Pacific islands [4]. Many of these species are well-known due to their medicinal properties and are used in the treatment of different diseases, especially those of the gastrointestinal, respiratory, genitourinary tracts and skin, as well as various infective and inflammatory diseases [5,6].

Phytochemical studies have shown that the genus *Plectranthus s.l.* is a rich source of phenolic compounds and terpenes. Phenolic compounds are represented by phenolic acids, their esters and flavonoids which are often methoxylated. Terpenes include mono- and sesquiterpenes, which are the main components of essential oils, diterpenes, and several triterpenes [5,7,8,9].

Most phytochemical studies have focused on diterpenes because of their structural diversity and promising biological activities. Abietane diterpenes are the most numerous and diverse group [5]. The present review provides a comprehensive and up-to-date overview of abietane diterpenes of *Plectranthus s.l.*, their structures, occurrence, and biosynthesis. The compound classification proposed by Abdel-Mogib et al. in 2002 [8] is updated and extended here.

Literature searches were performed in various databases (SciFinder, Science Direct, and Google Scholar) using the keywords “*Plectranthus*/*Coleus*/*Solenostemon*/*Pycnostachys/Tetradenia*/*Anisochilus*/*Aeollanthus*/*Capitanopsis*/*Thorncroftia*/*Neohyptis*/*Englerastrum* + diterpene + diterpenoid”.

## 2. Abietane Diterpenes of the Genus *Plectranthus s.l.*

Diterpenes encompass a heterogeneous group of natural compounds with a hydrocarbon skeleton with twenty carbon atoms corresponding to four building five-carbon isoprene units. Their ability to form rings which leads to the production of bicyclic, tricyclic, tetracyclic, and macrocyclic structures and the presence of oxygen functional groups, such as hydroxyls, carbonyls, and carboxyls, are responsible for a wide range of pharmacological properties [10].

More than 345 diterpenes from the abietane, beyerane, cembrane, clerodane, halimane, icetexane, kaurane, labdane, pimarane, and phyllocladane classes have been found in the genus *Plectranthus s.l.*, with almost 70% of them belonging to the abietane class [10,11,12,13,14]. Due to their numerous biological activities, including antioxidant, antimicrobial, antiprotozoal, cytotoxic and gastroprotective activity, abietanes are considered promising in the search for new drugs [15,16].

**Figure 1:**
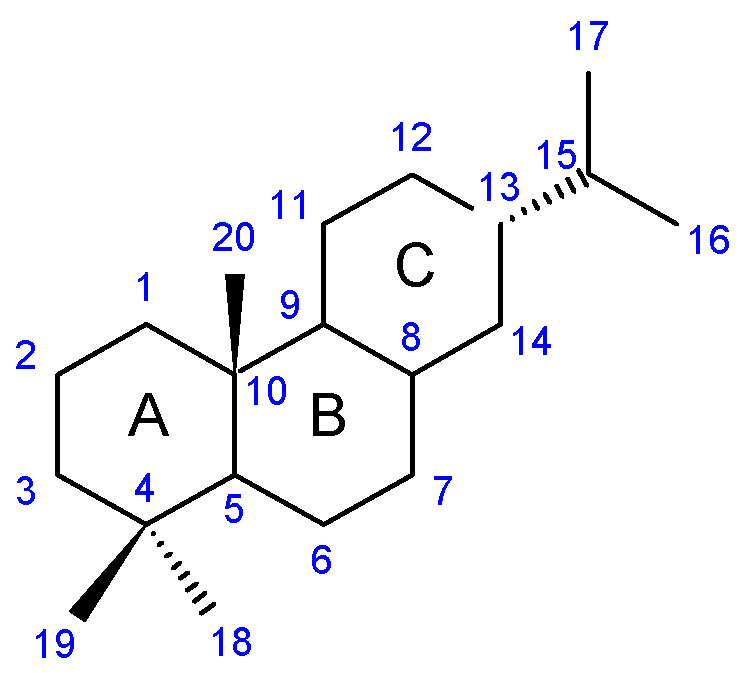
Hydrocarbon skeleton of abietane diterpenes [19,20].

Structurally, the basic abietane core skeleton is composed of a tricyclic perhydrophenanthrene of normal series, indicating that the methyl group (C20) attached to carbon C10 is always β-oriented. Two methyl groups (C18 and C19) are attached to carbon C4, and carbon C13 is substituted with an isopropyl group [10,11,17,18] (Figure 1).

Naturally occurring abietane diterpenes are always present in a highly oxygenated form. Hydroxylation and further oxidation (>CHOH → –C=O, –CH_3_ → –CH_2_OH → –CHO → –COOH) are among the most common events that take place in this group of compounds [21]. Hydroxyl groups often undergo esterification with aliphatic or aromatic acids, or etherification with alcohols. Formyl and carbonyl groups form hemiacetals or acetals by reacting with hydroxyls, and carboxyls react with hydroxyl groups to form esters or lactones [22]. The presence of oxygen-containing functional groups due to the processes mentioned above is also responsible for further modifications, such as rearrangements of the abietane skeleton, ring cleavages, or the complete aromatization of the ring system [23,24,25,26,27,28,29].

**Scheme 1:**
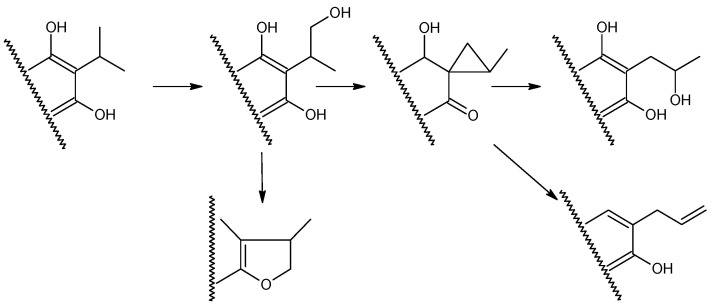
Metabolic pathway modifying the isopropyl side chain [23].

The isopropyl residue attached at C13 is not always present. Its catabolic pathway was first described by Schmid et al. in 1982; a 1-hydroxyprop-2-yl side chain is formed in the first step. Subsequent cyclisation may result in the formation of methyldihydrofuran or spirocyclopropane, leading to the formation of 2-hydroxyprop-1-yl or an allyl chain [23] (Scheme 1).

**Scheme 2:**
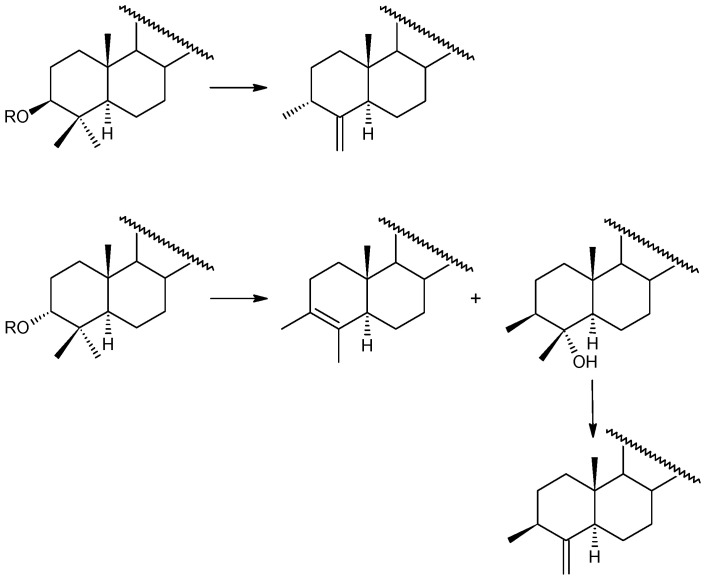
Biosynthesis of 18 and 19(4→3)-*abeo*-abietanes [24,25,30].

Another event occurring across different groups of abietanes is a migration of the methyl group from the position C4 to the neighbouring C3 position, resulting in the rearranged (4→3)-*abeo*-abietanes. Their biosynthesis arises from the 3-hydroxylated precursor (the hydroxyl may undergo other modifications). While 3β-oxyabietanes are considered precursors of 18(4→3)-*abeo*-abietanes, 3α-oxyabietanes are precursors of 19(4→3)-*abeo*-abietanes [24,25,30] (Scheme 2).

Abietanes found in the genus *Plectranthus s.l.* may be divided according to the structure of the ring C into two primary groups: abietanes with a quinoid arrangement of the ring C, and abietanes with an aromatic ring C; within these, other subgroups are distinguished, as proposed by Abdel-Mogib et al. in 2002 [8]. Abietanes with a quinoid arrangement of the ring C include royleanones (structures **1**–**66**), spirocoleons (structures **67**–**136**), quinone methides (structures **137**–**153**), vinylogous quinones (structures **154**–**164**), and 1,4-phenanthraquinones (structures **165**–**169**). Dimeric abietanes (structures **170**–**176**) consist of units with both quinoid and aromatic ring C, and abietanes with aromatic ring C are formed by hydroquinones (structures **177**–**217**), other phenolic abietanes (structures **218**–**236**), and non-phenolic abietanes (structures **237**–**243**).

### 2.1. Royleanones

**Figure 2:**
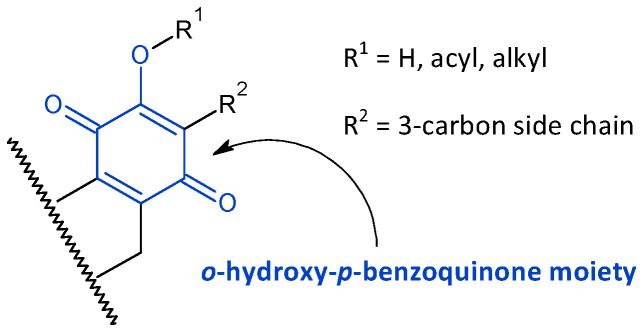
Royleanone skeleton.

Royleanones belong to the most numerous groups of abietane diterpenes in the genus *Plectranthus s.l.* They possess the characteristic 12-hydroxy-11,14-benzoquinone skeleton, also known as royleanone system [15] (Figure 2).

Royleanones were named after *Inula royleana*, where they were reported for the first time [15,31]. In 1958, Handa et al. described the yellow pigment in the roots of this plant. Further investigations showed a mixture of diterpenoid quinones composed of 6,7-dehydroroyleaone (**1**), royleanone (**2**), and 7-acetoxyroyleanone, which Edwards et al. named royleanones in 1962 [31].

In terms of biosynthesis, royleanones are considered oxidative derivatives of diterpenmonophenol ferruginol (**218**). It is assumed that the hydroxyl group at C12, as an activating group, directs hydroxylation to C11 (*o*-position) and C7 (*p*-position). Subsequently, the hydroxyl at C11 drives the entry of another hydroxyl substituent to C14 (*p*-position). The oxidation of *o*-hydroxyhydroquinone leads to the formation of *o*-hydroxy-*p*-benzoquinone [32].

Table 1 covers 66 compounds of the royleanone class (structures **1**–**66**), divided into seven subgroups according to the character of the 3-carbon side chain: royleanones with an isopropyl side chain (structures **1**–**26**), a 1-hydroxyprop-2-yl side chain (structures **27**–**29**), a 2-hydroxypropyl side chain (structures **30**–**39**), an allyl side chain (structures **40**–**55**), a 3-hydroxypropyl side chain (structures **56**–**58**), and a dihydropyran side chain (structure **59**). The group of *seco*-royleanones is mentioned separately, due to the significant rearrangement of their basic royleanone skeleton (structures **60**–**66**).

**Table 1:** Royleanones of the genus *Plectranthus s.l.*

(a) Royleanones with an Isopropyl Side Chain							
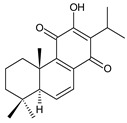 6,7-dehydroroyleanone (**1**) *Coleus* (Ethiopia) [33,34], *C. amboinicus* [35], *C. bishopianus* [36,37], *C. forsteri* ‘Marginatus’ [38], *C. grandidentatus* [39,40], *C. lanuginosus* [23], *C. maculosus* [41], *C. madagascariensis* [42,43]							
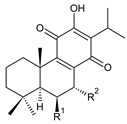							
**Compound Name (Numerical ID)**	**R^1^**	**R^2^**		**Source [References]**			
royleanone (**2**)	–H	–H		*C.* (Ethiopia) [33,34], *C. amboinicus* [35], *C. grandidentatus* [39,40], *C. maculosus* [41]			
6β-hydroxyroyleanone (**3**)	–OH	–H		*C. amboinicus* [35], *C. grandidentatus* [39,40], *C. maculosus* [41], *C. sanguineus* [28]			
horminone (syn. 7α-hydroxyroyleanone) (**4**)	–H	–OH		*C.* (Ethiopia) [33,34], *C. amboinicus* [35], *C. grandidentatus* [39,40], *C. hereroensis* [40,44,45], *C. madagascariensis* [46], *C. sanguineus* [28]			
7-*O*-formylhorminone (**5**)	–H	–OCHO		*C. sanguineus* [28]			
7α-acetoxyroyleanone (**6**)	–H	–OCOCH_3_		*C.* (Ethiopia) [34], *C. amboinicus* [35]			
6β,7α-dihydroxyroyleanone (**7**)	–OH	–OH		*C.* (Ethiopia) [33,34], *C.* (Rwanda) [29], *C. amboinicus* [35], *C. argentatus* [47], *C. bishopianus* [36,37], *C. grandidentatus* [40,48,49], *C. hereroensis* [50], *C. maculosus* subsp. *edulis* [26], *C. madagascariensis* [46], *C. sanguineus* [28]			
7α-acetoxy-6β-hydroxyroyleanone (**8**)	–OH	–OCOCH_3_		*C.* (Ethiopia) [33,34], *C. amboinicus* [35], *C. argentatus* [47], *C. grandidentatus* [39,40,48,49,51], *C. hadiensis* [16,52], *C. madagascariensis* [42,43,46], *C. sanguineus* [28]			
7α-acyloxy-6β-hydroxyroyleanone (mixture of esters) (**9**)	–OH	–palmityloxy –stearyloxy –oleyloxy *–n*-heptadecanoyloxy *–n*-pentadecanoyloxy –myristyloxy		*C. grandidentatus* [39,48,49]			
7α-formyloxy-6β-hydroxyroyleanone (**10**)	–OH	–OCHO		*C. argentatus* [47], *C. hadiensis* [16], *C. hereroensis* [50], *C. madagascariensis* [53], *C. sanguineus* [28]			
6β-formyloxy-7α-hydroxyroyleanone (**11**)	–OCHO	–OH		*C. argentatus* [47]			
6β-hydroxy-7α-methoxyroyleanone (**12**)	–OH	–OCH_3_		*C. bishopianus* [36,37]			
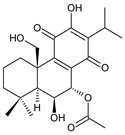 7α-acetoxy-6β,20-dihydroxyroyleanone (**13**) *C. amboinicus* [35]							
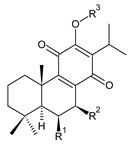							
**Compound Name (Numerical ID)**	**R^1^**	**R^2^**	**R^3^**	**Source [References]**			
taxoquinone (syn. 7β-hydroxyroyleanone) (**14**)	–H	–OH	–H	*C.* (Ethiopia) [33,34], *C. maculosus* [41]			
7β-acetoxyroyleanone (**15**)	–H	–OCOCH_3_	–H	*C. maculosus* [41]			
6β,7β-dihydroxyroyleanone (**16**)	–OH	–OH	–H	*C. forsteri* ‘Marginatus’ [38], *C. hadiensis* [52], *C. maculosus* [41], *C. madagascariensis* [42,43,54]			
7β-acetoxy-6β-hydroxyroyleanone (**17**)	–OH	–OCOCH_3_	–H	*C. forsteri* ‘Marginatus’ [38], *C. hadiensis* [52], *C. maculosus* [41], *C. madagascariensis* [54]			
6β,7β-dihydroxy-12-*O*-methylroyleanone (**18**)	–OH	–OH	–CH_3_	*C. maculosus* [41]			
7β-acetoxy-6β-hydroxy-12-*O*-methylroyleanone (**19**)	–OH	–OCOCH*_3_*	–CH_3_	*C. maculosus* [41]			
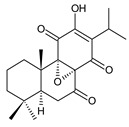 8α,9α-epoxy-7-oxoroyleanon (**20**) *C.* (Ethiopia) [34]							
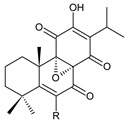							
**Compound Name (Numerical ID)**	**R**			**Source [References]**			
8α,9α-epoxy-6-deoxycoleon U-quinone (**21**)	–H			*C. maculosus* [41]			
8α,9α-epoxycoleon U-quinone (syn. 8α,9α-epoxy-8,9-dihydrocoleon U-quinone) (**22**)	–OH			*C. argentatus* [47], *C. maculosus* [41], *C. sanguineus* [28], *C. xanthanthus* [55]			
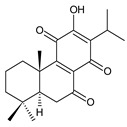 7-oxoroyleanone (syn. 7-ketoroyleanone) (**23**) *C.* (Ethiopia) [33,34]	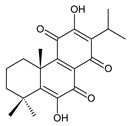 coleon U-quinone (**24**) *C. argentatus* [47], *C. forsteri* ‘Marginatus’ [56], *C. maculosus* [41], *C. madagascariensis* [46,54], *C. sanguineus* [28], *C. xanthanthus* [55]						
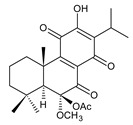 6β-acetoxy-6α-methoxy-7-oxoroyleanone (**25**) *C. maculosus* [41]			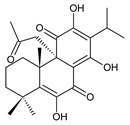 6,12,14-trihydroxy-9α-(2-oxopropyl)abieta-5,8(14),12-triene-7,11-dione (**26**) *C. grandidentatus* [51]				
**(b) Royleanones with a 1-Hydroxyprop-2-yl Side Chain**							
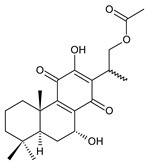 16-acetoxy-7α,12-dihydroxyabieta-8,12-diene-11,14-dione (**27**) *C. hereroensis* [40,45,57]	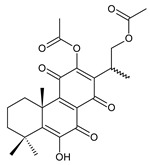 xanthanthusin H (**28**) *C. xanthanthus* [55]			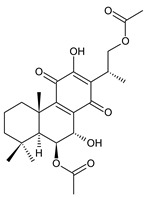 scutellarioidone B (**29**) *C. scutellarioides* [12]			
**(c) Royleanones with a 2-Hydroxypropyl Side Chain**							
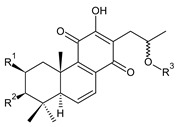							
**Compound Name (Numerical ID)**	**R^1^**	**R^2^**	**R^3^**	**Source [References]**			
plectranthone G (**30**)	–H	–OH	–COCH_3_	*C.* (Rwanda) [29]			
plectranthroyleanone A (**31**)	–OH	–H	–CH_3_	*C. engleri* [58]			
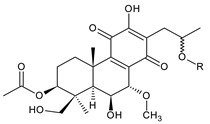							
**Compound Name (Numerical ID)**	**R**			**Source [References]**			
plectranthroyleanone B (**32**)	–CH_3_			*C. engleri* [58]			
plectranthroyleanone C (**33**)	–H			*C. engleri* [58]			
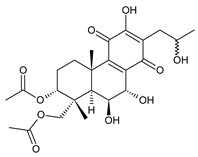 17(15→16)-*abeo*-3α,18-*bis*(acetoxy)-6β,7α,16ξ-trihydroxyroyleanone (**34**) *C. barbatus* var. *barbatus* [25]	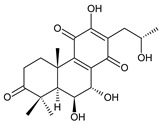 scutellarioidone A (**35**) *C. scutellarioides* [12,59]			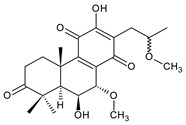 sincoetsin C (**36**) *C. scutellarioides* [59]			
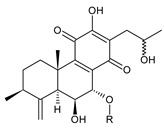							
**Compound Name (Numerical ID)**			**R**	**Source [References]**			
17(15→16),19(4→3)-*bis*(*abeo*)-6β,7α,16ξ-trihydroxyroyleanone (**37**)			–H	*C. barbatus* var. *barbatus* [25], *C. lanuginosus* [60]			
17(15→16),19(4→3)-*bis*(*abeo*)-6β, 16ξ-dihydroxy- 7α-methoxyroyleanone (**38**)			–CH_3_	*C. barbatus* var. *barbatus* [25]			
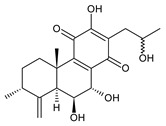 17(15→16),18(4→3)-*bis*(*abeo*)-6β,7α,16ξ-trihydroxyroyleanone (**39**) *C. lanuginosus* [60], *C. maculosus* subsp. *edulis* [26]							
**(d) Royleanones with an Allyl Side Chain**							
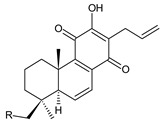							
**Compound Name (Numerical ID)**	**R**			**Source [References]**			
lanugon A (**40**)	–H			*C. lanuginosus* [23], *C. maculosus* subsp. *edulis* [26]			
lanugon B (**41**)	–OH			*C. lanuginosus* [23]			
lanugon C (**42**)	–OCHO			*C. lanuginosus* [23]			
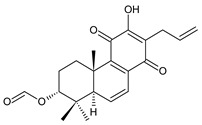 3α-formyloxylanugon A (**43**) *C. maculosus* subsp. *edulis* [26]				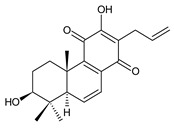 plectranthone F (**44**) *C.* (Rwanda) [29]			
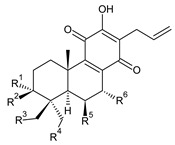							
**Compound Name (Numerical ID)**	**R^1^**	**R^2^**	**R^3^**	**R^4^**	**R^5^**	**R^6^**	**Source [References]**
7α,12-dihydroxy-17(15→16)-*abeo*-abieta-8,12,16- triene-11,14-dione (**45**)	–H	–H	–H	–H	–H	–OH	*C. hereroensis* [40,44]
6β,7α-dihydroxy(allyl)royleanone (**46**)	–H	–H	–H	–H	–OH	–OH	*C. sanguineus* [28]
plectranthone H (**47**)	–H	–OH	–H	–H	–OCOCH_3_	–H	*C.* (Rwanda) [29]
plectranthone I (**48**)	–H	–OH	–H	–H	–OCOCH_3_	–OH	*C.* (Rwanda) [29]
lanugon D (**49**)	–H	–H	–OCHO	–H	–OH	–OH	*C. lanuginosus* [23]
lanugon E (**50**)	–H	–H	–OH	–H	–OH	–OCH_2_CH_3_	*C. lanuginosus* [23]
3α-formyloxy-6β,7α-dihydroxy(allyl)royleanone (**51**)	–OCHO	–H	–H	–H	–OH	–OH	*C. maculosus* subsp. *edulis* [26]
18-acetoxy-3α-formyloxy-6β,7α-dihydroxy(allyl)royleanone (**52**)	–OCHO	–H	–H	–OCOCH_3_	–OH	–OH	*C. lanuginosus* [60]
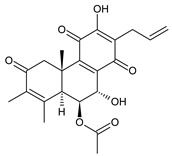 plectranthone J (**53**) *C.* (Rwanda) [29], *C. barbatus* [61]							
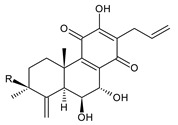							
**Compound Name (Numerical ID)**				**R**			**Source [References]**
17(15→16),18(4→3)-*bis*(*abeo*)-6β,7α,12-trihydroxyabieta-4(19),8,12,16-tetraene-11,14-dione (**54**)				–H			*C. maculosus* subsp. *edulis* [26]
3β-acetoxy-6β,7α,12-trihydroxy-17(15→16),18(4→3)-*bis*(*abeo*)-abieta-4(19),8,12,16-tetraene-11,14-dione (**55**)				–OCOCH_3_			*C. hereroensis* [39,45]
**(e) Royleanones with a 3-Hydroxypropyl Side Chain**							
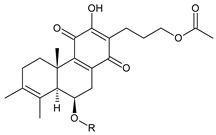							
**Compound Name (Numerical ID)**	**R**			**Source [References]**			
fredericon B (**56**)	–H			*C. fredericii* [62], *C. scutellarioides* [12]			
6-acetylfredericone B (**57**)	–COCH_3_			*C. scutellarioides* [12]			
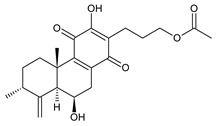 scutellarioidone D (**58**) *C. scutellarioides* [12]							
**(f) Royleanones with a Dihydropyrane Side Chain**							
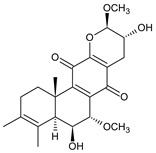 fredericone C (**59**) *C. fredericii* [62]							
**(g) *Seco*-Royleanones**							
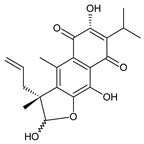 coleon A (**60**) *C. barbatus* var. *grandis* [63], *C.* aff. *Gracilis* [56], *C. igniarius* [64,65]							
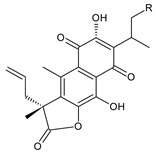							
**Compound Name (Numerical ID)**	**R**			**Source [References]**			
coleon A-lactone (**61**)	–H			*C. gracilis* [56], *C. maculosus* subsp. *Edulis* [26]			
16-acetoxycoleon A-lactone (**62**)	–OCOCH_3_			*C. maculosus* subsp. *Edulis* [26]			
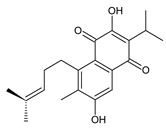 6,12-dihydroxysapriparaquinone (**63**) *C. maculosus* [41]							
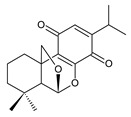 plectrabarbene (**64**) *C. barbatus* [66]	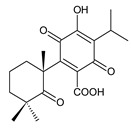 xanthanthusin E (**65**) *C. xanthanthus* [55]			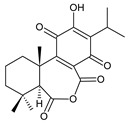 sanguinon A (**66**) *C. sanguineus* [28]			

**Scheme 3:**
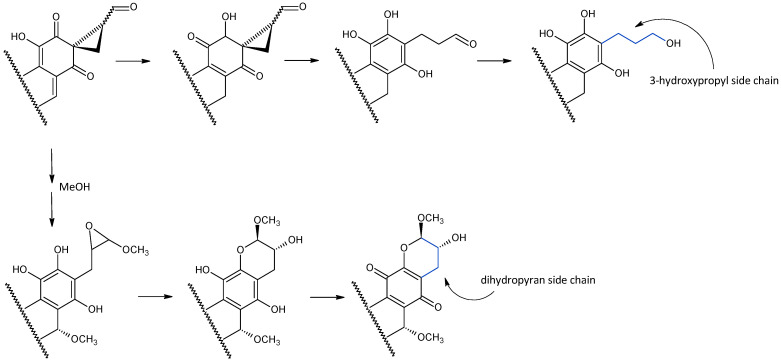
Biosynthesis of a 3-hydroxypropyl and a dihydropyrane side chain [62].

Dehydroroyleanone (**1**) and royleanone (**2**) are typical representatives of the royleanone class of diterpenes. They both bear an isopropyl side chain at C13, which is the most widespread type of short 3-carbon chain. However, the isopropyl side chain is not necessarily present and the position C13 can also be occupied by a 1-hydroxyprop-2-yl, 2-hydroxypropyl, allyl (Scheme 1), or unusual 3-hydroxypropyl and dihydropyran side chain. For example, fredericone B (**56**) possesses a 3-hydroxypropyl side chain. Its biosynthesis arises from the spirocyclopropane precursor fredericon A (**67**) and continues via a reductive opening of the cyclopropane ring. The subsequent reduction in the aldehyde group leads to the formation of primary alcohol. Fredericon C (**59**), arising from the same precursor, bears a dihydropyran chain formed by two nucleophilic openings of the cyclopropane and methoxyoxirane ring, respectively [62] (Scheme 3).

The ring B in royleanones often undergoes processes that result in double bond formation and hydroxy or keto substitution. A double bond between atoms C6 and C7 is a relatively common structural feature of royleanones, as well as of other abietanes. In addition to 6,7-dehydroroyleanone (**1**), lanugones A (**40**), B (**41**), and C (**42**) may also be mentioned as examples.

Oxygen-containing functional groups are often present at C6 and/or C7. A hydroxyl group can be attached at both C6 and C7. Compounds such as 6β-hydroxyroyleanone (**3**), horminone (**4**), and taxoquinone (**14**) are examples of royleanones with a monohydroxylated ring B, and 6β,7α-dihydroxyroyleanone (**7**) and 6β,7β-dihydroxyroyleanone (**16**) belong to royleanones with a dihydroxylated ring B. Besides the position of the hydroxyls, their orientation seems to be an important structural feature as well. While the C6 hydroxyl always has a β-orientation, the C7 hydroxyl can be both α- and β-oriented. A keto substitution has only ever been found at carbon C7, as demonstrated in the example of 7-oxoroyleanone (**23**) or coleon U-quinone (**24**).

Another oxidative modification observed in the ring B is 8,9-epoxidation, e.g., in 8,9-epoxycoleon U-quinone (**22**). In addition to the epoxy group, an uncommon double bond located between carbons C5 and C6 is present.

The ring A bears two methyl groups attached to carbon C4. However, these methyls can undergo oxidation—as present in plectranthroyleanones B (**32**) and C (**33**), or in 17(15→16)-*abeo*-3α,18-*bis*(acetoxy)-6β,7α,16ξ-trihydroxyroyleanone (**34**)—or can undergo migration from C4 to C3 (Scheme 2), leading to the formation of rearranged 18 or 19(4→3)-*abeo*-abietanes, as exemplified by 17(15→16),19(4→3)-*bis*(*abeo*)-6β,7α,16ξ-trihydroxyroyleanone (**37**), fredericone B (**56**), and scutellarioidone D (**58**). The oxidation can also take place at C3 or C2, resulting in hydroxy or keto derivatives, e.g., sincoetsin C (**36**), plectranthones F (**44**), G (**30**), H (**47**), I (**48**), and J (**53**), and plectranthroyleanone A (**31**).

The royleanone group also includes several compounds with less common structural features; 7α-acetoxy-6β,20-dihydroxyroyleanone (**13**), which possesses a hydroxylated C20 methyl group and a 6,12,14-trihydroxy-9α-(2-oxopropyl)abieta-5,8(14),12-triene-7,11-dione (**26**), with an unusual substitution with an 2-oxopropyl group at C9.

As stated above, royleanone diterpenes are often hydroxylated at different positions. Hydroxyls, especially those at the C6, C7, and C13 side chains, as well as at C3, C12, C18, and C19, undergo esterification. Although acetic and formic acids are the most common esterifying acid, Teixeira et al., 1997, reported the isolation and identification of 7α-acyloxy-6β-hydroxyroyleanone (**9**) from the acetone extract of *C. grandidentatus*. The compound was established as a mixture of esters with palmitic, stearic, oleic, *n*-heptadecanoic, *n*-pentadecanoic, and myristic acids [39].

**Scheme 4:**
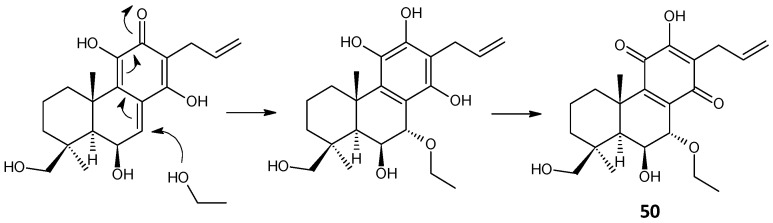
The formation of lanugon E (**50**) during the isolation process [23].

In addition to the esterification process, the alkylation of hydroxyl groups, leading to methylethers, has been described. The etherification of C6, C7, and C12 hydroxyls, and a 2-hydroxypropyl side chain has been reported. The compound 6β-acetoxy-6α-methoxy-7-oxoroyleanone (**25**) is an example, with an unusual arrangement of substituents bearing two modified hydroxyls at the same carbon atom. Lanugon E (**50**) is the only known example possessing an ethylated hydroxyl substituent. However, considering that the presence of this unusual substituent is explained by the nucleophilic addition of ethanol, used during the process of isolation (Scheme 4), lanugon E (**50**) is an artifact [23].

**Scheme 5:**
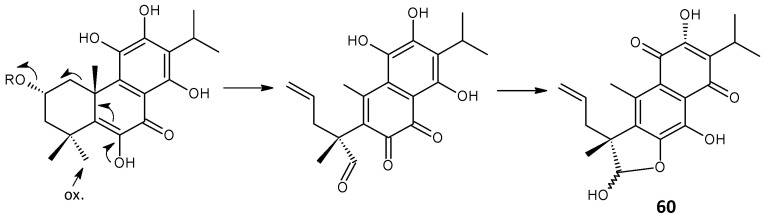
Biosynthesis of coleon A (**60**) [27].

Royleanones also include a small group of diterpenes whose basic skeleton is modified by cleavage of the ring A or B—*seco*-royleanones. Naphtoquinone coleon A (**60**) belongs to the 1,10-*seco*-abietanes. Its biosynthesis was elucidated in 1975 and arises from a hydroquinone precursor, in which hydroxylation at the C2 position enables the opening of the ring A. Oxidation of the C18 methyl group and tautomerization result in the formation of hemiacetal [27] (Scheme 5).

Another example with an uncommon structure is 4,5-*seco*-5,10-*friedo*-abietane 6,12-dihydroxysapriparaquinone (**63**), with a cleaved ring A and its C20 methyl group migrating from C10 to C5. Sanguinon A (**66**) is a 6,7-*seco*-abietane with an anhydride moiety in the ring B. This compound is considered a metabolite of coleons, with oxygen-containing groups at C6 and C7 [28].

### 2.2. Spirocoleons

Together with royleanones, spirocoleons are the most abundant class of abietane diterpenes of the genus *Plectranthus s.l.* Rüedi et al., 1972, postulated the biosynthesis of a 2-hydroxypropyl side chain through spirocyclopropyl-cyclohexendienon, which was only considered an unstable reactive intermediate [67]. However, by 1973, the first spirocoleons were already isolated [68,69].

**Figure 3:**
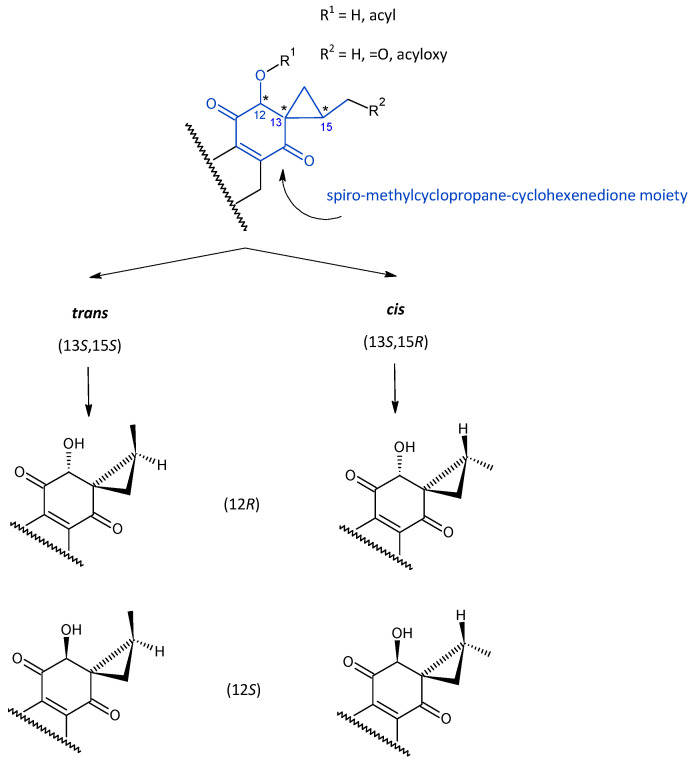
Spirocoleon skeleton and its stereochemistry [24,70].

Spirocoleons have a typical spiromethylcyclopropane-cyclohex-8-ene-11,14-dione arrangement of the ring C, hydroxylated at position C12. Three chiral centres at C12, C13, and C15 allow for eight possible stereoisomers, but only four of them have been detected in nature so far. Spirocoleons of the genus *Plectranthus s.l.* may be divided into two groups according to their configuration at C13 and C15. The first group includes *trans*-derivatives which have a (13*S*,15*S*)-configuration with a C=O and methyl group on opposite sides of the plane of the cyclopropane ring; the second group of *cis*-derivatives with a (13*S*,15*R*)-configuration bears a C=O and methyl on the same side of the reference plane of the cyclopropane ring [24,70] (Figure 3).

Table 2 includes 70 compounds (structures **67–136**), divided according to the absolute configuration of their ring C into three groups: compounds with an unspecified orientation of substituents in the cyclopropane ring (structure **67**), (13*S*,15*S*)-spirocoleons (structures **68–108**), and (13*S*,15*R*)-spirocoleons (structures **109–136**).

**Table 2:** Spirocoleons of the genus *Plectranthus s.l.*

(a) Compounds with the Unspecified Orientation in the Cyclopropane Ring							
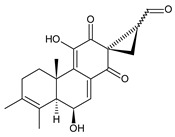 fredericon A (**67**) *C. fredericii* [62]							
**(b) Type (13*S*,15*S*)**							
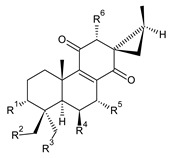							
**Compound Name (Numerical ID)**	**R^1^**	**R^2^**	**R^3^**	**R^4^**	**R^5^**	**R^6^**	**Source [References]**
lanugon K (**68**)	–H	–H	–H	–OH	–OCOCH_3_	–OH	*C. lanuginosus* [23], *C. scutellarioides* [12]
lanugon K’ (**69**)	–H	–H	–H	–OH	–OCHO	–OH	*C. lanuginosus* [23]
coleon Q (**70**)	–H	–H	–H	–OH	–OH	–OCOCH_3_	*C. caninus* [24]
12-*O*-desacetylcoleon Q (syn. 15-epilanugon F) (**71**)	–H	–H	–H	–OH	–OH	–OH	*C. harmandii* [71], *C. lanuginosus* [60]
7-*O*-acetyl-12-*O*-desacetyl-19-hydroxycoleon Q (**72**)	–H	–OH	–H	–OH	–OCOCH_3_	–OH	*C. lanuginosus* [60]
18-acetoxy-12-*O*-desacetylcoleon Q (**73**)	–H	–H	–OCOCH_3_	–OH	–OH	–OH	*C. harmandii* [71], *C. lanuginosus* [60]
6-*O*-acetyl-19-acetoxycoleon Q (**74**)	–H	–OCOCH_3_	–H	–OCOCH_3_	–OH	–OCOCH_3_	*C. autranii* [72], *C. garckeanus* [72]
12-*O*-desacetyl-6-*O*-acetyl-19-acetoxycoleon Q (**75**)	–H	–OCOCH_3_	–H	–OCOCH_3_	–OH	–OH	*C. harmandii* [71]
12-*O*-desacetyl-7-*O*-acetyl-19-acetoxycoleon Q (**76**)	–H	–OCOCH_3_	–H	–OH	–OCOCH_3_	–OH	*C. autranii* [72], *C. garckeanus* [72]
coleon R (**77**)	–OCOCH_3_	–H	–H	–OCOCH_3_	–OH	–OCOCH_3_	*C. caninus* [24], *C. comosus* [73]
12-*O*-desacetylcoleon R (**78**)	–OCOCH_3_	–H	–H	–OCOCH_3_	–OH	–OH	*C. barbatus* var. *barbatus* [25]
6,12-*bis*(*O*-desacetyl)coleon R (**79**)	–OCOCH_3_	–H	–H	–OH	–OH	–OH	*C. barbatus* var. *barbatus* [25]
coleon Y (**80**)	–OCOCH_3_	–H	–OCOCH_3_	–OH	–OH	–OH	*C. barbatus* var. *barbatus* [25], *C. lanuginosus* [60]
3-*O*-desacetyl-3-*O*-formylcoleon Y (**81**)	–OCHO	–H	–OCOCH_3_	–OH	–OH	–OH	*C. barbatus* var. *barbatus* [25], *C. lanuginosus* [60]
3,18-*bis*(*O*-desacetyl)3,18-*bis*(*O*-formyl)coleon Y (**82**)	–OCHO	–H	–OCHO	–OH	–OH	–OH	*C. lanuginosus* [60]
(13*S*,15*S*)-6β,7α,12α,19-tetrahydroxy-13β,16-cycloabiet-8-ene-11,14-dione (**83**)	–H	–OH	–H	–OH	–OH	–OH	*C. porcatus* [74]
(13*S*,15*S*)-6β,12α-*bis*(acetoxy)-3α-formyloxy-7α-hydroxy-13β,16-cycloabiet-8-ene-11,14-dione (**84**)	–OCHO	–H	–H	–OCOCH_3_	–OH	–OCOCH_3_	*C. maculosus* subsp. *edulis* [26]
(13*S*,15*S*)-3α,7α-diformyloxy-6β,12α-dihydroxy-13,16-cycloabiet-8-ene-11,14-dione (**85**)	–OCHO	–H	–H	–OH	–OCHO	–OH	*C. maculosus* subsp. *edulis* [26]
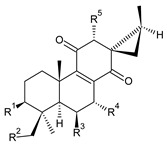							
**Compound Name (Numerical ID)**	**R^1^**	**R^2^**	**R^3^**	**R^4^**	**R^5^**	**Source [References]**	
12-*O*-desacetyl-7-*O*-acetyl-3β,19-*bis*(acetoxy)coleon Q (**86**)	–OCOCH_3_	–OCOCH_3_	–OH	–OCOCH_3_	–OH	*C. autranii* [72], *C. garckeanus* [72]	
3β-hydroxy-3-deoxybarbatusine (**87**)	–OH	–H	–OCOCH_3_	–OH	–OCOCH_3_	*C. barbatus* [75], *C. barbatus* var. *grandis* [76], *C. comosus* [77]	
ornatin G (**88**)	–OH	–H	–OCOCH_3_	–OH	–OH	*C. comosus* [77]	
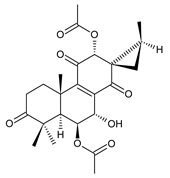 barbatusine (**89**) *C. barbatus* [68,75,78], *C. barbatus* var. *grandis* [76], *C. caninus* [24]			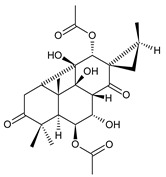 cyclobutatusine (**90**) *C. barbatus* [68,75,78]				
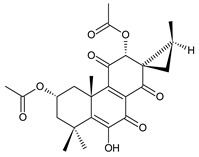 xanthanthusin I (**91**) *C. xanthanthus* [55]		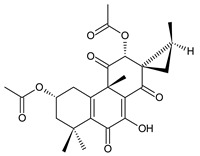 xanthanthusin J (**92**) *C. xanthanthus* [55]			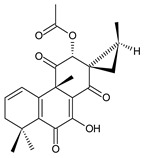 xanthanthusin K (**93**) *C. xanthanthus* [55]		
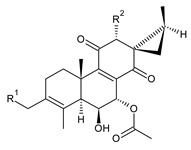							
**Compound Name (Numerical ID)**	**R^1^**	**R^2^**	**Source [References]**				
coleon M (**94**)	–H	–OCOCH_3_	*C. caninus* [24]				
coleon O (**95**)	–H	–OH	*C. autranii* [72], *C. barbatus* [79], *C. barbatus* var. *barbatus* [25], *C. caninus* [24], *C. garckeanus* [72], *C. lanuginosus* [24,60], *C. scutellarioides* [12,80]				
19-acetoxycoleon O (syn. syl-C) (**96**)	–OCOCH_3_	–OH	*C. autranii* [72,81]				
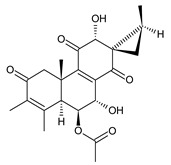 plectrin (**97**) *C. barbatus* [61,79]							
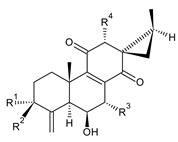							
**Compound Name (Numerical ID)**	**R^1^**	**R^2^**	**R^3^**	**R^4^**	**Source [References]**		
coleon G (**98**)	–CH_3_	–H	–OCOCH_3_	–OH	*C. lanuginosus* [60,69], *C. scutellarioides* [12]		
coleon J (**99**)	–CH_3_	–H	–OH	–OH	*C. lanuginosus* [60,69]		
coleon N (**100**)	–H	–CH_3_	–OCOCH_3_	–OCOCH_3_	*C. caninus* [24]		
7,12-*bis*(*O*-desacetyl)coleon N (**101**)	–H	–CH_3_	–OH	–OH	*C. barbatus* var. *barbatus* [25]		
12-*O*-desacetylcoleon N (**102**)	–H	–CH_3_	–OCOCH_3_	–OH	*C. barbatus* var. *barbatus* [25]		
7-desoxy-12-*O*-desacetyl-3-acetoxycoleon N (**103**)	–OCOCH_3_	–CH_3_	–H	–OH	*C. autranii* [72]		
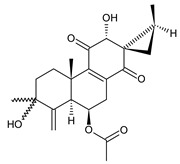 syl-B (**104**) *C. autranii* [81]	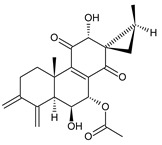 coleon Z (syn. syl-D) (**105**) *C. autranii* [72,81], *C. garckeanus* [72], *C. maculosus* subsp. *edulis* [26]				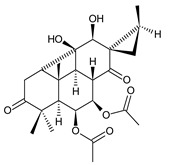 7β-acetyl-12-desacetoxycyclobutatusine (**106**) *C. barbatus* [78]		
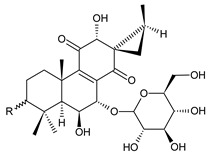							
**Compound Name (Numerical ID)**	**R**			**Source [References]**			
spirocoleon-7-*O*-β-D-glucoside (**107**)	–H			*C. scutellarioides* [59,82]			
3-hydroxyspirocoleon-7-*O*-β-D-glucoside (**108**)	–OH			*C. scutellarioides* [59]			
**(c) Type (13*S*,15*R*)**							
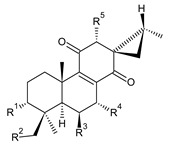							
**Compound Name (Numerical ID)**	**R^1^**	**R^2^**	**R^3^**	**R^4^**	**R^5^**	**Source [References]**	
lanugon F (**109**)	–H	–H	–OH	–OH	–OH	*C. lanuginosus* [23], *C. maculosus* subsp. *edulis* [26]	
lanugon G (**110**)	–H	–H	–OH	–OCHO	–OH	*C. lanuginosus* [23], *C. maculosus* subsp. *edulis* [26]	
lanugon H (**111**)	–H	–OH	–OH	–OCHO	–OH	*C. lanuginosus* [23]	
lanugon I (**112**)	–H	–OCHO	–OH	–OH	–OH	*C. lanuginosus* [23]	
lanugon J (**113**)	–H	–OCHO	–OH	–OCHO	–OH	*C. lanuginosus* [23]	
coleon P (**114**)	–H	–H	–OH	–OH	–OCOCH_3_	*C. caninus* [24], revision [70]	
12-*O*-desacetyl- 7-*O*-acetyl-19-acetoxycoleon P (**115**)	–H	–OCOCH_3_	–OH	–OCOCH_3_	–OH	*C. autranii* [72]	
plectranthone K (**116**)	–H	–H	–OH	–OCOCH_3_	–OH	*C.* (Rwanda) [29]	
(13*S*,15*R*)-6β-acetoxy-3α-formyloxy-7α,12α-dihydroxy-13β,16-cycloabiet-8-ene-11,14-dione (**117**)	–OCHO	–H	–OCOCH_3_	–OH	–OH	*C. maculosus* subsp. *edulis* [26]	
(13*S*,15*R*)-3α-formyloxy-6β,7α,12α-trihydroxy-13β,16-cycloabiet-8-ene-11,14-dione (**118**)	–OCHO	–H	–OH	–OH	–OH	*C. maculosus* subsp. *edulis* [26]	
(13*S*,15*R*)-3α,7α-diformyloxy-6β,12α-dihydroxy-13β,16-cycloabiet-8-ene-11,14-dione (**119**)	–OCHO	–H	–OH	–OCHO	–OH	*C. maculosus* subsp. *edulis* [26]	
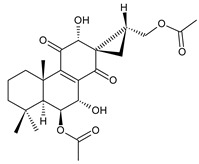 12-*O*-desacetyl-6-*O*-acetyl-17-acetoxycoleon P (syn. syl-A) (**120**) *C. autranii* [72,81], *C. garckeanus* [72]		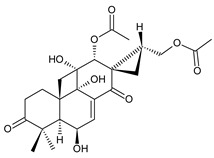 spiroscutelone A (**121**) *C. scutellarioides* [83]				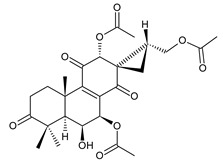 spiroscutelone B (**122**) *C. scutellarioides* [83]	
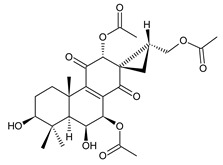 spiroscutelone C (**123**) *C. scutellarioides* [83]				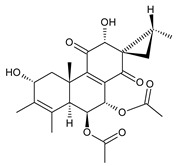 plectranthone L (**124**) *C.* (Rwanda) [29]			
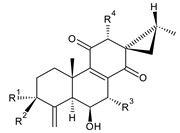							
**Compound Name (Numerical ID)**	**R^1^**	**R^2^**	**R^3^**	**R^4^**	**Source [References]**		
15-*epi*-coleon J (**125**)	–CH_3_	–H	–OH	–OH	*C. maculosus* subsp. *edulis* [26]		
7,12-*bis*(*O*-acetyl)-15-*epi*-coleon J (**126**)	–CH_3_	–H	–OCOCH_3_	–OCOCH_3_	*C. maculosus* subsp. *edulis* [26]		
7-*O*-formyl-12-*O*-acetyl-15-*epi*-coleon J (**127**)	–CH_3_	–H	–OCHO	–OCOCH_3_	*C. maculosus* subsp. *edulis* [26]		
7-*O*-acetyl-15-*epi*-coleon J (**128**)	–CH_3_	–H	–OCOCH_3_	–OH	*C. maculosus* subsp. *edulis* [26]		
12-*O*-acetyl-15-*epi*-coleon J (**129**)	–CH_3_	–H	–OH	–OCOCH_3_	*C. maculosus* subsp. *edulis* [26]		
7-*O*-formyl-15-*epi*-coleon J (**130**)	–CH_3_	–H	–OCHO	–OH	*C. maculosus* subsp. *edulis* [26]		
19(4→3)-*abeo*-3α,7α-*bis*(acetoxy)-6β,12α-dihydroxy-13β,16-cycloabieta-4(18),8-diene-11,14-dione (**131**)	–OCOCH_3_	–CH_3_	–OCOCH_3_	–OH	*C. maculosus* subsp. *edulis* [26]		
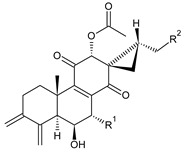							
**Compound Name (Numerical ID)**			**R^1^**	**R^2^**	**Source [References]**		
12-*O*-acetyl-15-*epi*-coleon Z (syn.12-*epi*-mon-A) (**132**)			–OCOCH_3_	–H	*C. monostachyus* [81,84], revision [70]		
12-*O*-acetyl-7-*O*-formyl-7-*O*-desacetyl-15-*epi*-coleon Z (syn. 12-*epi*-mon-B) (**133**)			–OCHO	–H	*C. monostachyus* [81,84], revision [70]		
12-*O*-acetyl-17-acetoxy-15-*epi*-coleon Z (syn. 12-*epi*-mon-C) (**134**)			–OCOCH_3_	–OCOCH_3_	*C. monostachyus* [81,84], revision [70]		
12-*O*-acetyl-17-formyloxy-15-*epi*-coleon Z (**135**)			–OCOCH_3_	–OCHO	*C. monostachyus* [81,84], revision [70]		
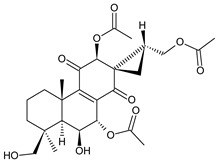 ornatin F (**136**) *C. comosus* [77]							

The first subgroup of spirocoleons is well represented by fredericone A (**67**) only. This compound bears a 1,1,2-tricarbonylcyclopropane moiety, which is responsible for its susceptibility to nucleophile-promoted rearrangements leading to the formation of fredericons B (**56**) and C (**59**) (Scheme 3) [62]. Lanugon K (**68**) and plectranthone K (**116**) are examples of the other two subgroups. They have an identical substitution in their spirocoleon basic skeleton and differ only in the orientation of the methyl group related to the plane of the cyclopropane ring. Lanugon K (**68**) belongs to the *trans*-derivatives, with a (13*S*,15*S*)-configuration, whereas plectranthone K (**116**) is a *cis*-derivative with a (13*S*, 15*R*)-configuration.

Another chiral centre is located at C12 and bears a hydroxyl group (the hydroxyl can undergo esterification), which can be α- or β-oriented. It was previously assumed that all spirocoleons are (12*R*)-isomers with an α-oriented hydroxyl [70]. Although most spirocoleons belong to the (12*R*)-isomers, the existence of 7β-acetyl-12-desacetoxycyclobutatusine (**106**) and ornatin F (**136**), isolated in 2007 and 2020, respectively, showed that (12*S*)-isomers with a β-orientation of the hydroxyl group exist in nature [77,78].

Spirocoleons undergo metabolic modifications, including oxidative processes or methyl migration similar to those described in royleanones, but there are several differences. Neither derivatives with a C7 monohydroxylated ring B nor with methylated hydroxyls have been found. On the other hand, a keto substitution at C6 has been described, e.g., in xanthanthusin J (**92**).

Several spirocoleons with uncommon structural features have been isolated. Xanthanthusines J (**92**) and K (**93**) are examples of rearranged 20(10→9)-*abeo*-abietanes. Cyclobutatusin (**90**), 7β-acetyl-12-desacetoxycyclobutatusine (**106**), and spiroscutelone A (**121**) are abietanes with a cyclobutane ring, formed as a result of C1–C11 and C20–C11 linkages, respectively. Coleon Z (**105**) contains two exocyclic methyliden groups at C3 and C4.

Most of the abietane diterpenes exist as aglycones, but spirocoleons have also been found as glycosides. Spirocoleon 7-*O*-β-D-glucoside (**107**) and 3-hydroxyspirocoleon 7-*O*-β-D-glucoside (**108**) are the only known abietanes of the genus *Plectranthus s.l.* that bind a molecule of sugar.

### 2.3. Quinone Methides

**Figure 4:**
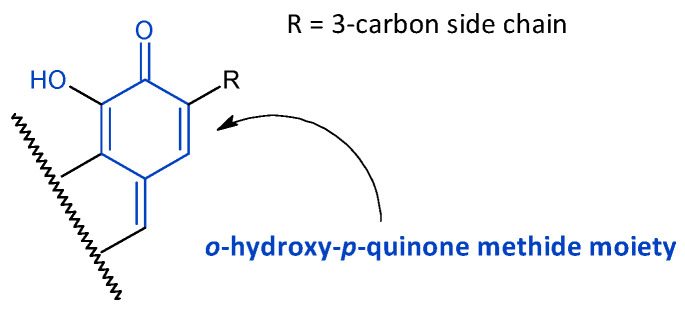
Quinone methide skeleton.

Quinone methides are abietane diterpenes with an *o*-hydroxy-*p*-quinone methide substructure. This structural moiety comprises a cyclohexa-9(11),13-diene ring C with a keto group at C12, a hydroxyl group at C11, and a C7 methine group (Figure 4). Quinone methides are considered reactive compounds. While the *o*-hydroxyl enhances their reactivity through an intramolecular hydrogen bond, the extended conjugation, together with a conformation of terpenoids, has a stabilizing effect [85].

Quinone methides belong to smaller groups of abietane diterpenes and include the 17 compounds (structures **137–153**) listed in Table 3.

**Table 3:** Quinone methides of the genus *Plectranthus s.l.*

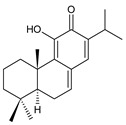 11-hydroxy-12-oxo-7,9(11),13-abietatriene (**137**) *P. elegans* [86]		
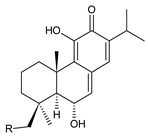		
**Compound Name (Numerical ID)**	**R**	**Source [References]**
19-senecioyloxytaxodone (**138**)	(CH_3_)_2_C=CH–COO–	*P. purpuratus* [14]
19-isovaleroyloxytaxodone (**139**)	(CH_3_)_2_CH–CH_2_–COO–	*P. purpuratus* [14]
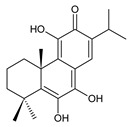 5,6-didehydro-7-hydroxytaxodone (**140**) *C. barbatus* [87]		
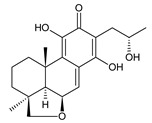 lanugon L (**141**) *C. lanuginosus* [23]	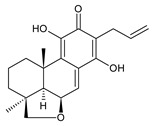 lanugon M (**142**) *C. lanuginosus* [23]	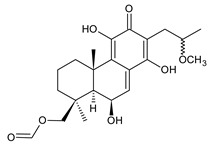 lanugon N (**143**) *C. lanuginosus* [23]
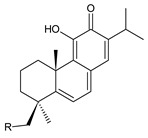		
**Compound Name (Numerical ID)**	**R**	**Source [References]**
parviflorone A (**144**)	(CH_3_)_2_C=CH–COO–	*C. australis* [88], *P. lucidus* [16], *P. purpuratus* [14], *P. purpuratus* subsp. *purpuratus* [16], *P. strigosus* [89]
11-hydroxy-19-(3-methylbutanoyloxy)-5,7,9 (11),13-abietatetraen-12-one (**145**)	(CH_3_)_2_CH–CH_2_–COO–	*P. purpuratus* [14]
parviflorone B (**146**)	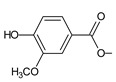	*C. australis* [88], *P. strigosus* [89]
parviflorone C (**147**)	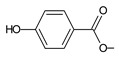	*C. australis* [88], *P. pururatus* subsp. *tongaensis* [16], *P. strigosus* [89]
parviflorone E (**148**)	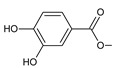	*C. australis* [88], *P. purpuratus* subsp. *tongaensis* [16], *P. strigosus* [89], *P. verticillatus* [90]
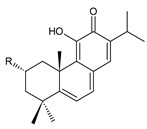		
**Compound Name (Numerical ID)**	**R**	**Source [References]**
parviflorone D (**149**)	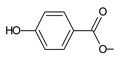	*C. australis* [88], *P. ecklonii* [16,42,43,91,92], *P. lucidus* [16], *P. strigosus* [89,93]
parviflorone F (**150**)	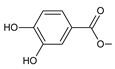	*C. australis* [88], *P. ecklonii* [16,91], *P. strigosus* [89], *P. verticillatus* [90]
parviflorone G (**151**)	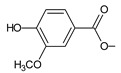	*P. strigosus* [89]
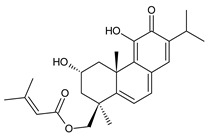 parviflorone H (**152**) *P. strigosus* [89]	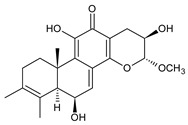 fredericone D (**153**) *C. fredericii* [62]	

Structurally, 11-hydroxy-12-oxo-7,9(11),13-abietatriene (**137**) is the simplest example of the quinone methide class of compounds. Further modifications include hydroxylation at positions C2, C6, C7, C14, and C19, oxidation of the C13 isopropyl side chain leading to the formation of a 2-hydroxypropyl or allyl chain (Scheme 1), as well as another double bond between C5 and C6 that extends the conjugation system.

The hydroxylation at C6 is one of the most common structural features within the abietane diterpenes of the genus *Plectranthus s.l.* While the C6 hydroxyl of royleanones and spirocoleons is always β-oriented, in quinone methides, the hydroxyl at C6 can have both an α- and β-orientation. The compounds 19-senecioyloxytaxodone (**138**) and 19-isovaleroyloxytaxodone (**139**) are examples with an α-oriented hydroxyl group at C6.

**Scheme 6:**
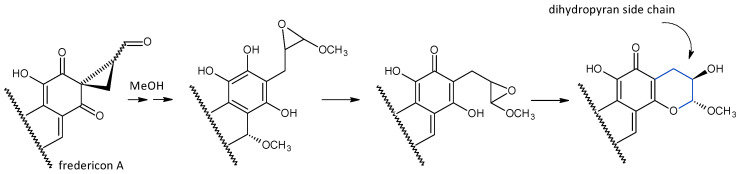
Biosynthesis of the dihydropyrane side chain of fredericon D (**153**) [62].

In addition to isopropyl, 2-hydroxypropyl, and allyl side chains, cyclisation producing dihydropyran has been detected in fredericone D (**153**). This compound arises from spirocoleon fredericone A (**67**) by similar a mechanism to the royleanone fredericon C (**59**) (Scheme 3) [62]. The cyclisation of the fredericon D (**153**) side chain is explained in Scheme 6.

Lanugones L (**141**) and M (**142**) have a rare 6,19-epoxy moiety; lanugon N (**143**) is an example that possesses hydroxyls modified by both esterification with formic acid and methylation. However, esterification with unusual aliphatic and aromatic acids is more frequent in this group of diterpenes. Parviflorones (**144–152**) are red-coloured quinone methides [89] that are separately or simultaneously substituted with hydroxyl groups at positions C2 and C19, which form esters with aliphatic senecioic acid or aromatic *p*-hydroxybenzoic, protocatechuic, or vanillic acid. Isovaleric acid is another esterifying acid established in the structure of 11-hydroxy-19-(3-methylbutanoyloxy)-5,7,9(11),13-abietatetraen-12-one (**145**).

### 2.4. Vinylogous Quinones

**Figure 5:**
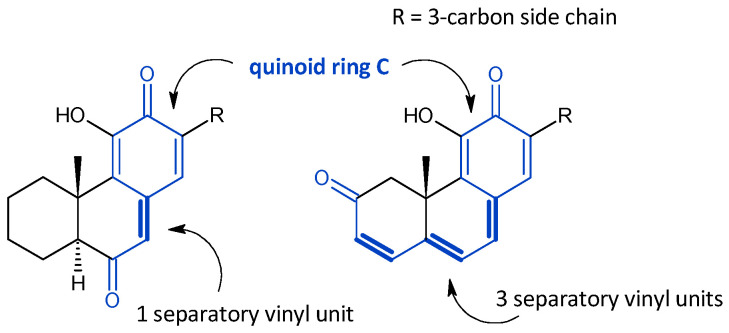
Skeleton of vinylogous quinones.

Vinylogous quinones, also known as extended quinones, represent a group of abietanes that have a cyclohexa-9(11),13-diene ring C with a keto group at C12 and a hydroxyl group at C11. The second keto group is separated by one or three vinyl units –CH=CH– from the quinoid ring C (Figure 5). Due to the formation of a conjugated system, an electronic effect of this functional group may be propagated along the molecule and may manifest in remote positions of the molecule [94].

This relatively small group includes 11 compounds (structures **154**–**164**), listed in Table 4. Further classification is based on the number of separatory units. While structures **154**–**162** belong to the first subgroup of vinylogous quinones with one separatory unit, structures **163** and **164** are vinylogous quinones with three separatory units.

**Table 4:** Vinylogous quinones of the genus *Plectranthus s.l.*

(a) Vinylogous Quinones with One Separatory Unit			
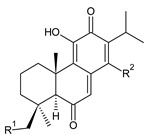			
**Compound Name (Numerical ID)**	**R^1^**	**R^2^**	**Source [References]**
taxodione (**154**)	–H	–H	*C. barbatus* [87]
19-senecioyloxytaxodione (**155**)	(CH_3_)_2_C=CH–COO–	–H	*P. purpuratus* [14]
19-isovaleroyloxytaxodione (**156**)	(CH_3_)_2_CH–CH_2_–COO–	–H	*P. purpuratus* [14]
14-hydroxytaxodione (**157**)	–H	–OH	*C. grandidentatus* [95]
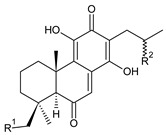			
**Compound Name (Numerical ID)**	**R^1^**	**R^2^**	**Source [References]**
17(15→16)-*abeo*-16ξ-acetoxy-11,14-dihydroxy-7,9(11),13-abietatriene-6,12-dione (**158**)	–H	–OCOCH_3_	*C. maculosus* subsp. *edulis* [26]
17(15→16)-*abeo*-11,14,16ξ -trihydroxy-7,9(11),13-abietatriene-6,12-dione (**159**)	–H	–OH	*C. maculosus* subsp. *edulis* [26]
lanugon P (**160**)	–OCHO	–OH	*C. lanuginosus* [23]
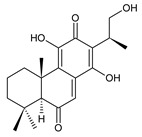 lanugon O (161) *C. lanuginosus* [23], *C. maculosus* subsp. *edulis* [26]		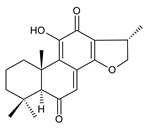 lanugon Q (162) *C. lanuginosus* [23]	
**(b) Vinylogous Quinones with Three Separatory Units**			
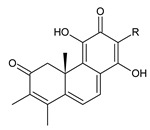			
**Compound Name (Numerical ID)**	**R**	**Source [References]**	
coleon E (**163**)	–CH_2_CH(OH)CH_3_	*C.* (Rwanda) [29], *C. barbatus* [61,67,79,96]	
coleon F (**164**)	–CH_2_CH=CH_2_	*C.* (Rwanda) [29], *C. barbatus* [61,97]	

The isopropyl side chain is not necessarily present in this group of substances. Methyldihydrofuran, 1-hydroxyprop-2-yl, allyl, and 2-hydroxypropyl side chains have been reported as well. In addition to a 3-carbon side chain, hydroxylation takes place at carbons C14 and C19. While the C19 hydroxyl can form esters with formic, senecioic, or isovaleric acid, the hydroxyl group of the 2-hydroxypropyl side chain has been found to form an ester with acetic acid.

In terms of structure, taxodione (**154**) is the simplest example of a vinylogous quinone with one separatory vinyl unit and a keto group at C6. Its hydroxylated and esterified derivatives 19-senecioyloxytaxodione (**155**) and 19-isovaleroyloxytaxodione (**156**), as well as the hydroxylated derivative 14-hydroxytaxodione (**157**), have been identified in the genus *Plectranthus s.l.* The α-methyldihydrofuran stage arising from the metabolization of an isopropyl side chain (Scheme 1) occurs relatively rarely, e.g., in lanugon Q (**162**). The second subgroup of vinylogous quinones with three separatory vinyl units and a keto group at C2 is represented by coleons E (**163**) and F (**164**).

### 2.5. 1,4-Phenathraquinones

**Figure 6:**
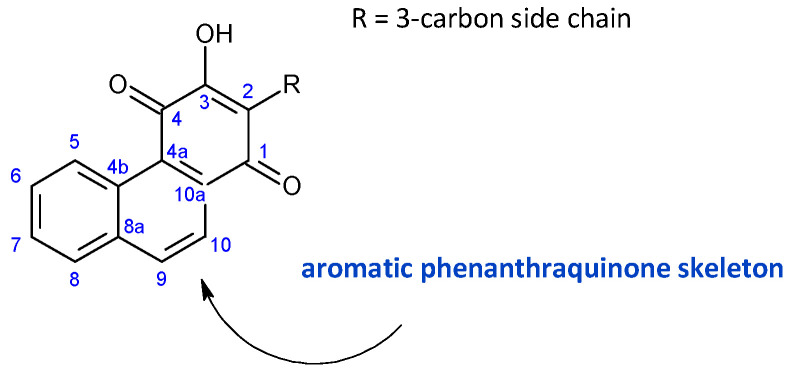
Skeleton of 1,4-phenanthraquinones [29].

A small group of 1,4-phenanthraquinone diterpenes has an aromatic tricyclic phenanthrene-1,4-dione skeleton with a hydroxyl attached at C3 and a 3-carbon side chain at C2 (having different numbering, as depicted in Figure 6).

The isolation of diterpenes with an extensive conjugated system of four double bonds spreading through the A, B, and C rings, such as coleon E (**163**) and F (**164**), led to the assumption that such compounds may be precursors of diterpenes with an aromatic phenathrene skeleton [27,29]. The first phenthraquinone diterpenes were obtained from the unknown *Plectranthus s.l.* species from Rwanda in 1984 [98]. In their biosynthesis, hydroxylation at the C6 and C7 positions, together with further hydroxylation at C3 and/or C2, seems to be essential for the formation of a conjugated system and following complete aromatisation. The exact sequence of reactions leading to the production of phenathraquinone diterpenes has not yet been described. Although the reason for these modification reactions has not been revealed, these extensive oxygenation and dehydrogenation processes probably precede the degradation of the ring system [29]. This group of abietanes is represented by five compounds (structures **165**–**169**), summarized in Table 5.

**Table 5:** Phenanthraquinones of the genus *Plectranthus s.l.*

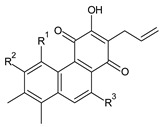				
**Compound Name (Numerical ID)**	**R^1^**	**R^2^**	**R^3^**	**Source [References]**
plectranthone A (**165**)	–CH_3_	–H	–H	*C.* (Rwanda) [29,98]
plectranthone C (**166**)	–H	–H	–H	*C.* (Rwanda) [29,98]
plectranthone D (**167**)	–H	–H	–CH_3_	*C.* (Rwanda) [29,98]
plectranthone E (**168**)	–H	–OH	–H	*C.* (Rwanda) [29]
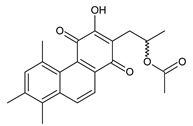 plectranthone B (**169**) *C.* (Rwanda) [29,98]				

This group of diterpenes includes only plectranthones A (**165**), B (**169**), C (**166**), D (**167**), and E (**168**). All of these compounds are substituted with a hydroxyl group at C3 and a methyl group at C7 and C8. They differ from each other in their substitutions at carbons C5, C6, and C10. Except for plectranthone B (**169**), possessing a 2-hydroxypropyl side chain esterified with acetic acid, phenanthraquinones bear allyl side chains attached at C2.

### 2.6. Dimeric Diterpenes

Dimeric diterpenes represent relatively rare group of diterpenes and are composed of two twenty-carbon diterpenic units linked through two C–C bonds, ester bonds, ether bonds, or a ring moiety [22]. In the genus *Plectranthus s.l.,* seven compounds (structures **170–176**) of this class have been found. They are listed in Table 6.

**Table 6:** Dimeric diterpenes of the genus *Plectranthus s.l.*

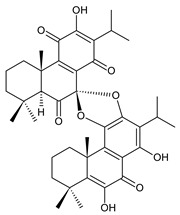 grandidone A (**170**) *C. grandidentatus* [48,49,95], *C. hereroensis* [95], *C. sanguineus* [28]		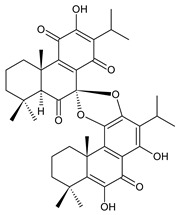 7-*epi*-grandidone A (**171**) *C. grandidentatus* [95], *C. sanguineus* [28]
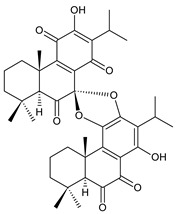 grandidone B (**172**) *C. amboinicus* [95], *C. grandidentatus* [95], *C. sanguineus* [28]		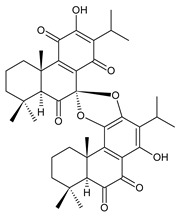 7-*epi*-grandidone B (**173**) *C. amboinicus* [95], *C. grandidentatus* [95], *C. sanguineus* [28]
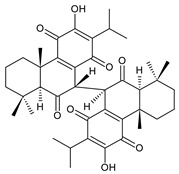 grandidone C (**174**) *C. grandidentatus* [39,95]	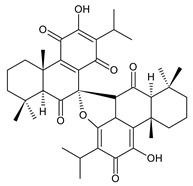 grandidone D (**175**) *C. grandidentatus* [39,95]	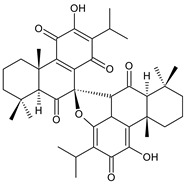 7-*epi*-grandidone D (**176**) *C. grandidentatus* [39,95]

In 1981, seven dimeric abietanes were isolated. Grandidones A (**170**) and B (**172**) are acetals of 6,7-dioxoroyleanone and coleon U (**183**) and coleon V (**204**), respectively. The linkage between diterpenic units is a result of the reaction between the C7 keto group of 6,7-dioxoroyleanone and two hydroxyl groups at C11′ and C12′ of coleon U (**183**) and coleon V (**204**), respectively. Grandidone C (**174**) is a dimer of *bis*(6-oxoroyleanone), whose diterpenic units are linked by a C–C bond between carbon atoms C7 and C7′. Considering that 6-oxoroyleanone can easily tautomerize to 14-hydroxytaxodione, it is supposed that grandidone D (**175**) forms via *bis*(14-hydroxytaxodione) by an intramolecular nucleophilic attack of the C14 (or C14′) hydroxyl at carbon C7′ (or C7), leading to the formation of a spirodihydrofuran linkage moiety. While grandidones are (7*S*)-derivatives, their corresponding *epi*-grandidones belong to the 7*R*-series [95].

### 2.7. Hydroquinones

Together with royleanones and spirocoleons, hydroquinones belong to the most numerous groups of abietane diterpenes of the genus *Plectranthus s.l.* They are characterised by an aromatic ring C substituted with hydroxyl groups at C11, C12, and C14.

**Figure 7:**
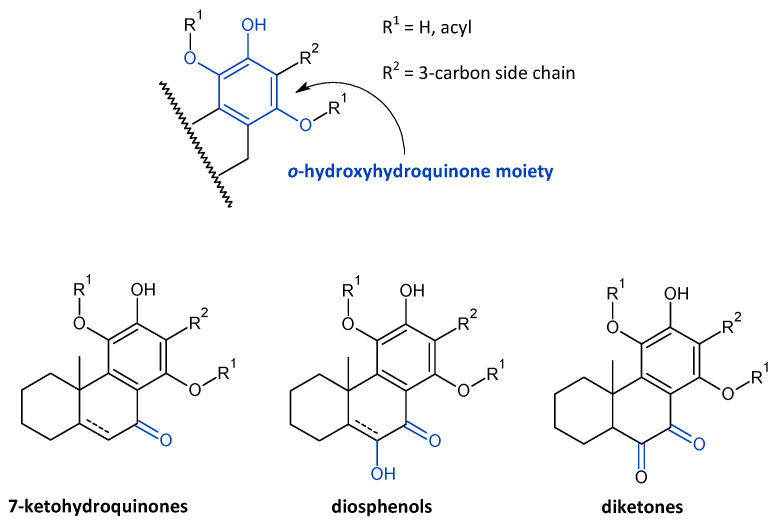
Hydroquinone skeleton and the substitution of the B ring.

In terms of further classification, substitution with oxygen-containing functional groups at C6 and C7 is important. The former includes the monosubstituted 7-ketohydroquinones, and the latter 6,7-disubstituted derivatives are traditionally divided into diosphenols (6-hydroxy-7-ketohydroquinones) and diketones (the 6,7-diketohydroquinones; Figure 7).

Hydroquinones are the largest group of abietanes with an aromatic ring C, with 41 compounds (structures **177**–**217**) divided according to further substitution of the ring B into 7-ketohydroquinones (structures **177**–**179**), diosphenols (**180**–**202**), and diketones (**203**–**214**). Due to a significant rearrangement of the basic hydroquinone skeleton, 5(4→3)-*abeo*-acylhydroquinones (structures **215**–**216**) and *seco*-hydroquinone (structure **217**) are mentioned separately. All these compounds are summarized in Table 7.

**Table 7:** Hydroquinones of the genus *Plectranthus s.l.*

(a) 7-Ketohydroquinones						
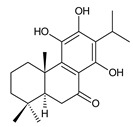 demethylinuroyleanol (**177**) *C. maculosus* [41]						
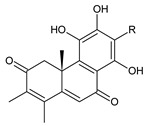						
**Compound Name (numerical ID)**				**R**	**Source [References]**	
plectrinone A (**178**)				–CH_2_CH(OH)CH_3_	*C.* (Rwanda) [29], *C. barbatus* [61,99]	
plectrinone B (**179**)				–CH_2_CH=CH_2_	*C.* (Rwanda) [29], *C. barbatus* [61]	
**(b) Diosphenols**						
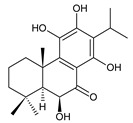 5,6-dihydrocoleon U (**180**) *C. argentatus* [47], *C. sanguineus* [28]					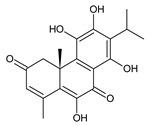 coleon B (**181**) *C. igniarius* [64,100]	
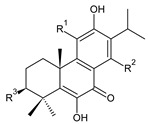						
**Compound Name (Numerical ID)**	**R^1^**	**R^2^**	**R^3^**	**Source [References]**		
coleon S (**182**)	–OH	–OH	–OH	*C. caninus* [101]		
coleon U (**183**)	–OH	–OH	–H	*C. amboinicus* [35], *C. argentatus* [47], *C. forsteri* ‘Marginatus’ [56], *C. grandidentatus* [40,48,49,51,95], *C. hereroensis* [50], *C. maculosus* subsp. *edulis* [26], *C. sanguineus* [28], *C. xanthanthus* [55]		
14-*O*-acetylcoleon U (**184**)	–OH	–OCOCH_3_	–H	*C. grandidentatus* [102]		
coleon U 11-acetate (**185**)	–OCOCH_3_	–OH	–H	*C. xanthanthus* [55]		
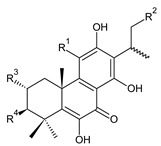						
**Compound Name (Numerical ID)**	**R^1^**	**R^2^**	**R^3^**	**R^4^**	**Source [References]**	
11,16-*bis*(*O*-acetoxy)coleon U (**186**)	–OCOCH_3_	–OCOCH_3_	–H	–H	*C. xanthanthus* [55]	
coleon C (**187**)	–OH	–OH	–H	–H	*C.* (North Madagascar) [103], *C. barbatus* var. *barbatus* [25], *C. lanuginosus* [23], *C. maculosus* subsp. *edulis* [26,104], *C. monostachyus* [84]	
(15*R*)-2α-formyloxy-coleon C (**188**)	–OH	–OH	–OCHO	–H	*C. monostachyus* [84]	
(15*S*)-2α-acetoxycoleon C (**189**)	–OH	–OH	–OCOCH_3_	–H	*C.* (Rwanda) [29]	
16-*O*-acetylcoleon C (**190**)	–OH	–OCOCH_3_	–H	–H	*C. barbatus* var. *barbatus* [25], *C. maculosus* subsp. *edulis* [26], *C. xanthanthus* [55]	
coleon H (**191**)	–OH	–OH	–H	–OCOCH_3_	*C.* (Rwanda) [29], *C. comosus* [77], *C. lanuginosus* [60,105], *C. monostachyus* [84], revision [106]	
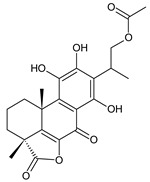 lactone derivative of 16-*O*-acetylcoleon C (**192**) *C. maculosus* subsp. *edulis* [26]						
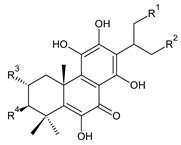						
**Compound Name (Numerical ID)**	**R^1^**	**R^2^**	**R^3^**	**R^4^**	**Source [References]**	
coleon L (**193**)	–OCOCH_3_	–OH	–H	–OCOCH_3_	*C. comosus* [77], *C. lanuginosus* [60,106]	
coleon W (**194**)	–OCOCH_3_	–OH	–H	–H	*C. autranii* [72], *C. barbatus* var. *barbatus* [25], *C. garckeanus* [72], *C. hereroensis* [50]	
16(or 17)-*O*-acetylcoleon W (**195**)	–OCOCH_3_	–OCOCH_3_	–H	–H	*C. autranii* [72], *C. garckeanus* [72]	
2α-acetoxycoleon W (**196**)	–OCOCH_3_	–OH	–OCOCH_3_	–H	*C. scutellarioides* [12,83,107]	
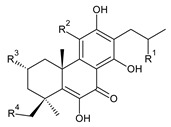						
**Compound Name (Numerical ID)**	**R^1^**	**R^2^**	**R^3^**	**R^4^**	**Source [References]**	
lanugon S (**197**)	–OH	–OH	–H	–OCHO	*C. lanuginosus* [23]	
13-desisopropyl-13-(2-hydroxypropyl)coleon U (**198**)	–OH	–OH	–H	–H	*C. maculosus* subsp. *edulis* [26]	
xanthanthusin F (**199**)	–OH	–OCOCH_3_	–OCOCH_3_	–H	*C. xanthanthus* [55]	
xanthanthusin G (**200**)	–OCH(CH_3_)_2_	–OCOCH_3_	–OCOCH_3_	–H	*C. xanthanthus* [55]	
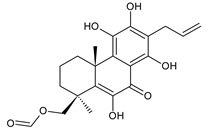 lanugon R (**201**) *C. lanuginosus* [23]				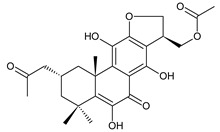 scutellarioidone C (**202**) *C. scutellarioides* [12]		
**(c) Diketones**						
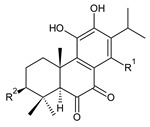						
**Compound Name (Numerical ID)**			**R^1^**	**R^2^**	**Source [References]**	
coleon T (**203**)			–OH	–OH	*C. caninus* [101]	
coleon V (**204**)			–OH	–H	*C. amboinicus* [35], *C. argentatus* [47], *C. barbatus* var. *barbatus* [25], *C. grandidentatus* [95], *C. hereroensis* [50], *C. maculosus* [41], *C. sanguineus* [28]	
14-*O*-formylcoleon V (**205**)			–OCHO	–H	*C. hereroensis* [50]	
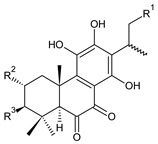						
**Compound Name (Numerical ID)**	**R^1^**	**R^2^**	**R^3^**	**Source [References]**		
coleon D (**206**)	–OH	–H	–H	*C.* (North Madagascar) [103], *C. barbatus* var. *barbatus* [25], *C. lanuginosus* [23,60], *C. maculosus* subsp. *edulis* [26,108]		
(15S)-2α -acetoxycoleon D (**207**)	–OH	–OCOCH_3_	–H	*C.* (Rwanda) [29]		
16-*O*-acetylcoleon D (**208**)	–OCOCH_3_	–H	–H	*C. barbatus* var. *barbatus* [25], *C. maculosus* subsp. *edulis* [26]		
coleon I (**209**)	–OH	–H	–OCOCH_3_	*C.* (North Madagascar) [103], *C.* (Rwanda) [29], *C. lanuginosus* [60,105], revision [106]		
coleon I’ (**210**)	–OH	–H	–OCHO	*C.* (North Madagascar) [103], revision [106]		
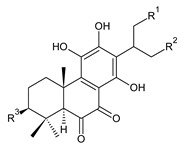						
**Compound Name (Numerical ID)**	**R^1^**			**R^2^**	**R^3^**	**Source [References]**
coleon K (**211**)	–OCOCH_3_			–OH	–OCOCH_3_	*C. lanuginosus* [60,105], revision [106]
coleon X (**212**)	–OCOCH_3_			–OH	–H	*C. autranii* [72], *C. garckeanus* [72]
16(or 17)-*O*-acetylcoleon X (**213**)	–OCOCH_3_			–OCOCH_3_	–H	*C. garckeanus* [72]
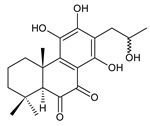 13-desisopropyl-13-(2-hydroxypropyl)coleon V (**214**) *C. maculosus* subsp. *edulis* [26]						
**(d) 5(4** **→3)-*Abeo*-Acylhydroquinones**						
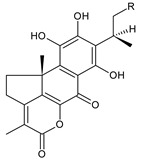						
**Compound Name (Numerical ID)**				**R**	**Source [References]**	
edulon A (**215**)				–OCOCH_3_	*C. maculosus* subsp. *edulis* [26,109,110]	
desacetyledulon A (**216**)				–OH	*C. maculosus* subsp. *edulis* [26]	
**(e) *Seco*-Hydroquinones**						
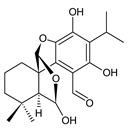 cariocal (**217**) *C. barbatus* [111]						

**Scheme 7:**
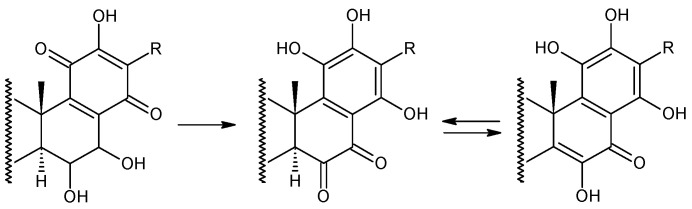
Biosynthesis of hydroquinones [27].

Their biosynthesis was postulated in 1975 and arises from 6,7-dihydroxyroyleanones that have undergone redox processes and tautomerization. The reduction in the ring C results in the formation of a hydroquinone ring C, whereas the oxidation of the ring B, hydroxylated at the C6 and C7 positions, leads to 6,7-diketones. The *trans*-A/B-6,7-diketones are very unstable and can easily convert into *trans*-A/B-diosphenols [27] (Scheme 7). As such, the tautomer pairs coleon U (**183**) and coleon V (**204**), or coleon S (**182**) and coleon T (**203**) may be mentioned.

Coleon W (**194**) and coleon X (**212**) are tautomeric acylhydroquinones that bear unusual side chains. In addition to isopropyl, 1-hydroxyprop-2-yl, allyl, and 2-hydroxypropyl side chains, several hydroquinones with a 1,3-dihydroxyprop-2-yl side chain—which is always esterified—have been isolated. Scutellarioidone C (**202**) is another example of a hydroquinone, with an uncommon β-methyldihydrofuran side chain linked to its abietane skeleton at the C12 and C13 positions, with an β-methyl group hydroxylated and esterified with acetic acid. The methylation of hydroxyls, as is often present in royleanones, does not occur in the hydroquinone class. Although, xanthanthusin G (**200**) is an exception, bearing a C13 2-hydroxypropyl side chain alkylated with an isopropyl group.

The hydroxyl functions at C2, C3, C18, C19, C11, and C14 often undergo esterification, especially with acetic or formic acid, e.g., xanthanthusin F (**199**), lanugon R (**201**) or coleon K (**211**). Such compounds are also known as acylhydroquinones.

**Scheme 8:**
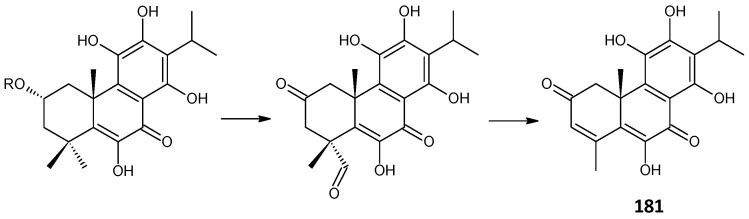
Biosynthesis of coleon B (**181**) [27].

Coleon B (**181**) is a yellow-coloured hydroquinone with a conjugated system of double bonds, possessing only one methyl group attached at C4. This 18-nor-abietane was isolated together with *seco*-royleanone coleon A from *C. igniarius* in 1963 [64]. Their biosynthesis arises from the same precursor. However, in the case of coleon B (**181**), the C2 hydroxyl oxidizes to ketone and the C18 methyl group is oxidatively cleaved [27] (Scheme 8).

**Scheme 9:**
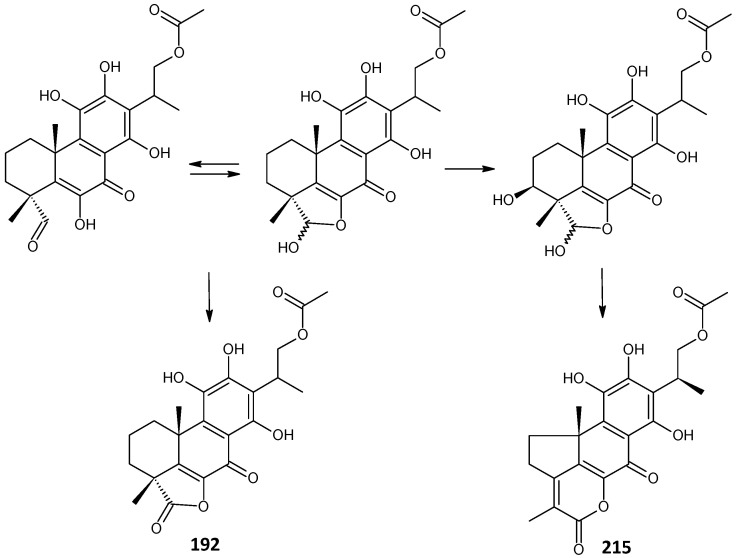
Biosynthesis of lactone derivative of 16-*O*-acetylcoleon C (**192**) and edulon A (**215**) [26].

The oxidation of C18 geminal methyl may result in the production of a lactone ring, as occurs in the lactone derivative of 16-*O*-acetylcoleon C (**192**). This γ-enol lactone is considered crucial in the understanding of the formation of 5(4→3)-*abeo*-acylhydroquinones such as edulon A (**215**). In 1987, Kunzle et al. postulated that lactone derivative of 16-*O*-acetylcoleon C (**192**) is formed from the aldehyde/lactol precursor. The hydroxylation of the precursor at C3 leads to the contraction of the ring A and the formation of edulon A (**215**) with a stabile α-pyrone system [26] (Scheme 9).

Cariocal (**217**) is another example of a hydroquinone diterpene with a modified tricyclic abietane skeleton. It is a highly oxidized 6,7-*seco*-abietane possessing both an acetal and hemiacetal function at C20 and C6 [111].

### 2.8. Other Phenolic Abietanes

**Figure 8:**
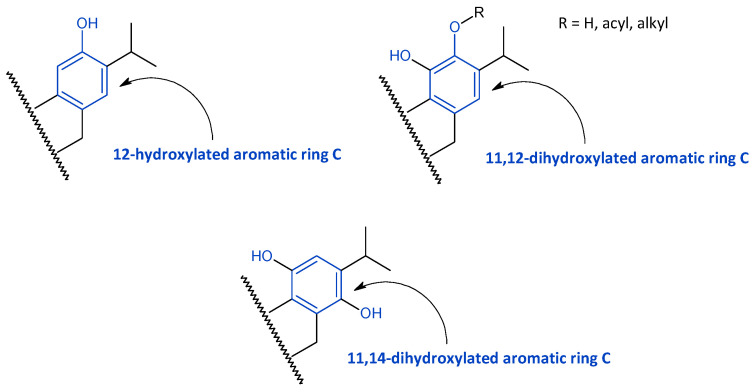
Skeletons of other phenolic abietanes and their substitution with hydroxyl groups.

This class of abietanes includes compounds with an aromatic ring C, differing in the degree of its hydroxylation. The substitution with one hydroxyl group at C12 (12-hydroxylated aromatic abietanes) or two hydroxyl groups at C11 and C12 (11,12-dihydroxylated aromatic abietanes), or C11 and C14 (11,14-dihydroxylated aromatic abietanes), may be present (Figure 8).

Nineteen compounds of phenolic abietanes (structures **218**–**236**) are listed in Table 8. They are divided, according to the number and position of hydroxyls at their ring C, into 12-hydroxylated aromatic abietanes (structures **218**–**221**), 11,12-dihydroxylated aromatic abietanes (structures **222**–**233**), 11,14-dihydroxylated aromatic abietanes (structures **234**), and *seco*-abietanes (structures **235**–**236**).

**Table 8:** Other phenolic abietanes of the genus *Plectranthus s.l.*

(a) 12-Hydroxylated Aromatic Abietanes					
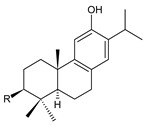					
**Compound Name (Numerical ID)**	**R**		**Source [References]**		
ferruginol (**218**)	–H		*C. barbatus* [13,112,113]		
hinokiol (**219**)	–OH		*P. strigosus* [93]		
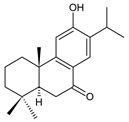 sugiol (**220**) *C. barbatus* [66,112,114]			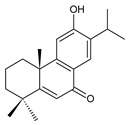 5,6-dehydrosugiol (**221**) *C. barbatus* [112]		
**(b) 11,12-Dihydroxylated Aromatic Abietanes**					
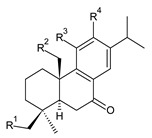					
**Compound Name (Numerical ID)**	**R^1^**	**R^2^**	**R^3^**	**R^4^**	**Source [References]**
11-hydroxysugiol (**222**)	–H	–H	–OH	–OH	*C. hadiensis* [115]
11,20-dihydroxysugiol (**223**)	–H	–OH	–OH	–OH	*C. hadiensis* [115]
19-*O*-senecioylester of 11,19-dihydroxysugiol (**224**)	(CH_3_)_2_C=CH–COO–	–H	–OH	–OH	*P. purpuratus* [14]
19-*O*-isovaleroylester of 11,19-dihydroxysugiol (**225**)	(CH_3_)_2_CH–CH_2_–COO–	–H	–OH	–OH	*P. purpuratus* [14]
plectranthol A (**226**)	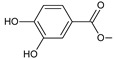	–H	–OH	–OH	*P. verticillatus* [90]
plectranthol B (**227**)	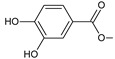	–H	–OH	(CH_3_)_2_C=CH–COO–	*P. verticillatus* [90]
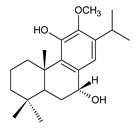 7α,11-dihydroxy-12-methoxy-8,11,13-abietatriene (**228**) *P. elegans* [86]		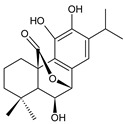 6β- hydroxycarnosol (**229**) *C. barbatus* [113]			
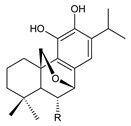					
**Compound Name (Numerical ID)**	**R**		**Source [References]**		
20-deoxocarnosol (**230**)	–H		*C. barbatus* [87,113]		
6α,11,12-trihydroxy-7β,20-epoxy-8,11,13-abietatriene (**231**)	–OH		*C. barbatus* [87]		
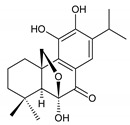 carnosolon (**232**) *C. amboinicus* [35], *C. hadiensis* [115], *C. madagascariensis* [46]		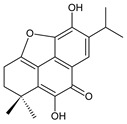 1,11-epoxy-6,12-dihydroxy-20-norabieta-1(10),5,8,11,13-pentaen-7-one (**233**) *C. hadiensis* [115]			
**(c) 11,14-Dihydroxylated Aromatic Abietane**					
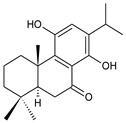 11,14-dihydroxy-8,11,13-abietatrien-7-one (**234**) *C. barbatus* [66,114]					
**(d) *Seco*-Abietanes with Phenolic Ring C**					
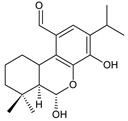 *seco*-abietane 1 (**235**) *C. barbatus* [30]			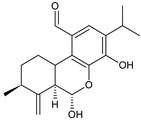 *seco*-abietane 2 (**236**) *C. barbatus* [30]		

As shown in Table 8, all of these compounds bear an isopropyl side chain at C13. Other hydroxyl groups—which can undergo further esterification—have been found at C3, C6, C7, C19, and C20. The C19 hydroxyl, as well as the C12 hydroxyl, may be esterified with aliphatic acids such as senecioic or isovaleric acid, or aromatic protocatechuic acid. In addition to esterification, the C12 hydroxyl can undergo methylation. The keto group may be present at C7, and several derivatives possess a double bond between C5 and C6. Even though these modifications are similar to those described above, within this group, several compounds with unusual structural features have been reported.

Plectranthol B (**227**) is esterified with both aromatic protocatechuic acid and aliphatic senecioic acid. The compound 6β-hydroxycarnosol (**229**) has a lactone ring between C20 and C7. The compound 1,11-epoxy-6,12-dihydroxy-20-norabieta-1(10),5,8,11,13-pentaen-7-one (**233**) is an unusual diterpene without a C20 methyl group, bearing an ether bridge between C1 and C11 and a double bond located between C1 and C10. Although the β-orientation of the hydroxyl group attached at C6 seems to be a characteristic feature of royleanones and spirocoleons of *Plectranthus s.l.*, 6α,11,12-trihydroxy-7β,20-epoxy-8,11,13-abietatriene (**231**) shows that 6α-hydroxylated derivatives also exist. *Seco*-abietane 1 (**235**) and *seco*-abietane 2 (**236**) are two remarkable 6,7-*seco*-abietanes with an aromatic ring C that are considered to be related to cariocal (**217**). While *seco*-abietane 1 (**235**) bears two methyl groups at C18 and C19 attached to carbon C4, *seco*-abietane 2 (**236**) is a rearranged 19(4→3)-*abeo*-abietane.

### 2.9. Non-Phenolic Aromatic Abietanes

The last class of abietane diterpenes occurring in the genus *Plectranthus s.l.* includes a small group of compounds with an aromatic ring C without any substitution with hydroxyl groups. The seven compounds (structures **237**–**243**) belonging to this small class are listed in Table 9.

**Table 9:** Non-phenolic abietanes of the genus *Plectranthus s.l.*

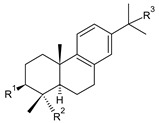				
**Compound Name (Numerical ID)**	**R^1^**	**R^2^**	**R^3^**	**Source [References]**
dehydroabietane (abietatriene) (**237**)	–H	–CH_3_	–H	*C. barbatus* [87]
4-*epi*-triptobenzene L (**238**)	–OH	–CH_2_OH	–H	*C. harmandii* [71]
dehydroabietic acid (**239**)	–H	–COOH	–H	*C. harmandii* [71]
15- hydroxydehydroabietic acid (**240**)	–H	–COOH	–OH	*C. harmandii* [71]
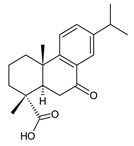 7-oxodehydroabietic acid (**241**) *C. harmandii* [71]	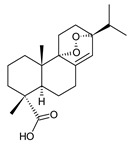 9α-13α-epidioxyabiet-8(14)-en-18-oic acid (**242**) *C. harmandii* [71]			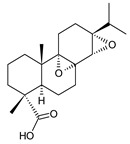 8α,9α,13α,14α-diepoxyabietan-18-oic acid (**243**) *C. harmandii* [71]

Dehydroabietane (**237**), also known as abietatriene, is an example of a compound with the simplest structure. However, this tricyclic hydrocarbon easily undergoes further modifications—especially the oxidation of the C18 methyl group. The compound 4-*epi*-triptobenzene L (**238**) is an example of a derivative hydroxylated at C3 and C18. The compound 7-oxodehydroabietic acid (**241**) is an example of a C18-diterpenic acid with a keto substitution at C7, and 15-hydroxydehydroabietic acid (**240**) bears an unusual 2-hydroxyprop-2-yl side chain. The oxidative processes may also affect the aromatic ring C as demonstrated on the examples of 9α-13α-epidioxyabiet-8(14)-en-18-oic acid (**242**)—which has a rare endoperoxide moiety—and epoxidic diterpene 8α,9α,13α,14α-diepoxyabietan-18-oic acid (**243**).

## 3. Occurrence and Isolation of Abietane Diterpenes

Phytochemical studies on African plants belonging to the families Lamiaceae and Fabaceae conducted by Eugster et al. in the 1950s led to the isolation of new leaf pigments stored in red-coloured leaf glands. Based on the presence of similar glands in African *Plectranthus* species, Eugster et al. suspected that this genus might also be a source of similar compounds, which led to the isolation of two new diterpenoid pigments, coleon A (**60**) and B (**181**), found in *C. igniarius* in 1963 [64,65,100]. This discovery triggered an extensive study of diterpenes of the genus *Plectranthus s.l*.

Most of the abietane diterpenes included in this review were isolated from the aerial parts due to their characteristic accumulation in leaf glands. However, several compounds were also isolated from the roots (*C. hereroensis* [40,44,57], *C. maculosus* [41]) or the whole plants (*C. engleri* [58], *C. grandidentatus* [39,40,48,49], *C. hadiensis* [52], *C. madagascariensis* [53], *P. ecklonii* [42,43,92], *P. strigosus* [93]).

The plant material was mostly extracted by maceration using different organic solvents such as diethyl ether, dichloromethane, chloroform, acetone, ethyl acetate, benzene, methanol, and ethanol. Some extracts were prepared by maceration with a mixture of two or three different solvents, e.g., the extract from the roots of *C. maculosus* with dichloromethane and methanol (1:1) [41], or the extract from the aerial parts of *C. madagascariensis* with hexane, dichloromethane, and acetone (2:2:1) [46]. Another modification of the process of maceration concerns using different solvents with increasing polarity. The air-dried leaves and stems of *C. lanuginosus* were extracted successively with hexane and diethyl ether [60]. The air-dried leaves of *C. comosus* were macerated with hexane followed by ethanol [77]. Extraction of plant material at room temperature under sonication has also been described in several studies, e.g., the aerial parts of *C. forsteri* ‘Marginatus’ [38] and *C. madagascariensis* [54] were extracted with methanol in an ultrasonic bath. Moreover, the leaves of *C. bishopianus* were extracted with hot methanol [36,37], the aerial parts of *C. maculosus* subsp. *edulis* with hot benzene [104], and the aerial parts of *C. lanuginosus* were extracted by percolation with diethyl ether [23].

Matias et al., 2019, prepared extracts from *C. madagascariensis* with different solvents (acetone, methanol, and supercritical CO_2_) by maceration, ultrasound-assisted extraction, and supercritical fluid extraction. Maceration and ultrasound-assisted extraction with methanol and acetone were more effective than supercritical fluid extraction with supercritical CO_2_ in term of yields. The authors explained these results by the ability of alcohol to disrupt the plant cell walls. It was also shown that using polar extraction solvents resulted in higher extraction yields [53].

Column chromatography (especially using silica gel, Sephadex LH-20, and polyamide as stationary phase) is the most common and convenient method for the isolation of abietane diterpenes. Many pure compounds were obtained directly by separation using column chromatography. However, this method is often combined with other chromatographic methods. Ndjoubi et al., 2021, obtained the abietanes from *C. madagascariensis* by silica gel column chromatography, selected fractions were successively purified by column chromatography with silica gel or Sephadex LH-20 as stationary phase, and semi-preparative HPLC was used to obtain coleon U-quinone (**24**) [46].

Mesquita et al., 2021, isolated abietane diterpenes from *C. comosus* using different chromatographic methods: column chromatography (using silica gel and Sephadex LH-20 as stationary phase), semi-preparative HPLC (using C18 column and normal phase column), and flash chromatography (using silica gel) [77].

In addition to chromatographic methods, crystallization is another method suitable for the isolation of abietane diterpenes. Kubínová et al., 2013, isolated 6,7-dehydroroyleanone (**1**) by crystallization from the essential oil obtained by steam-distillation of the fresh aerial parts of *C. forsteri* ‘Marginatus’ [38].

## 4. Biological Activity of Abietane Diterpenes

Many abietanes produced by the genus *Plectranthus s.l.* exhibit significant biological activities proving their medicinal potential. Although antitumoral activity is the most studied [116], antimicrobial, antiprotozoal, and gastroprotective activities have also been reported [15].

### 4.1. Antitumoral and Cytotoxic Activity

Abietane diterpenes, especially of the royleanone class, have shown cytotoxic effects on different cell lines. The cytotoxic and DNA-damaging activity of royleanone (**2**), horminone (**4**), and 7-acetoxyroyleanone were tested on human colon carcinoma cells Caco-2 and human hepatoma cells HepG2. All of the royleanones tested reduced the viability of cells and induced breaks in the DNA strand in both cell lines. However, an increased level of apoptotic nuclei comparable to camptothecin was observed only in HepG2 cells treated with horminone (**4**), and 7-acetoxyroyleanone [117]. 

Royleanone (**2**), horminone (**4**), 7α-acetoxyroyleanone (**6**), 7-ketoroyleanone (**23**), ferruginol (**218**), and sugiol (**220**) were tested for their cytotoxicity on the human pancreatic MIAPaCa-2 and melanoma MV-3 tumor cell lines. While all of these compounds acted cytotoxically on MIAPaCa-2, only horminone (**4**), 7α-acetoxyroyleanone (**6**), 7-ketoroyleanone (**23**), and sugiol (**220**) also had cytotoxic effects in the MV-3 cell line. 7α-Acetoxyroyleanone (**6**) was the most active against both the MIAPaCa-2 and MV-3 cell lines [118].

The abietane diterpenes 6,7-dehydroroyleanone (**1**), 7α-acetoxy-6β-hydroxyroyleanone (**8**), 6β,7β-dihydroxyroyleanone (**16**), and parviflorone D (**149**) acted cytotoxically against the human leukemia cell line CCRF-CEM and the lung adenocarcinoma A549. While 7α-acetoxy-6β-hydroxyroyleanone (**8**) exhibited the strongest cytotoxicity against CCRF-CEM cells, the strongest cytotoxic effect towards A549 was observed for parviflorone D (**149**). These compounds were also found to exhibit anticancer effects in the primary H7PX glioma cell line. Parviflorone D (**149**) demonstrated the greatest cytotoxic activity [42,43].

6,7-Dehydroroyleanone (**1**), 6β,7α-dihydroxyroyleanone (**7**), and 6β-hydroxy-7α-methoxyroyleanone (**12**) showed remarkable cytotoxic activity against the MCF-7 mammalian breast cancer cell line, greater than the effect caused by cisplatin used as a positive control [37].

6,7-Dehydroroyleanone (**1**) was also investigated for its ability to inhibit cell growth in different cell lines. Comparison of the IC_50_ values showed slight selectivity against different cancer cell lines. 6,7-Dehydroroyleanone (**1**) was more effective against the leukemia cells MOLT-3 and HL-60 than against the non-small-cell lung carcinoma cells NCI-H460, NCI-H460/R, and A549. Moreover, a growth inhibitory effect was also observed in human embryonal bronchial epithelial cells MRC-5 [119].

The cytotoxic activity of 6β,7α-dihydroxyroyleanone (**7**), 7α-acetoxy-6β-hydroxyroyleanone (**8**), 7α-formyloxy-6β-hydroxyroyleanone (**10**), and coleon U (**183**) was assayed in metastatic breast cancer MDA-MB-231, estrogen-dependent breast carcinoma MCF-7, colorectal carcinoma HCT116, normal human embryonal bronchial epithelial cells MRC-5, non-small cell lung carcinoma NCI-H460, and the multidrug-resistant non-small cell lung carcinoma cell line with P-glycoprotein overexpression NCI-H460/R. Except for coleon U (**183**), the tested abietane diterpenes did not act cytotoxically on MDA-MB-231 cells. On the other hand, all of them affected MCF-7 cells. The lowest antiproliferative effect shown by 6β,7α-dihydroxyroyleanone (**7**) was significantly increased by the structural modifications at C7 found in 7α-formyloxy-6β-hydroxyroyleanone (**10**), 7α-acetoxy-6β-hydroxyroyleanone (**8**), and coleon U (**183**). Royleanones 6β,7α-dihydroxyroyleanone (**7**) and 7α-acetoxy-6β-hydroxyroyleanone (**8**) exhibited high selectivity based on comparison of the growth inhibitory effects for NCI-H460 and MCR-5 cells. The abietanes tested similarly inhibited the growth of NCI-H460 and its multidrug-resistant variant NCI-H460/R. In this series of royleanones the higher lipophilicity of 7α-substituent led to the stronger cytotoxicity. The presence of an electron-donating substituent at C6 and/or C7 and log *P* between 2 and 5 also seem to be important for the structure–activity relationships [53].

6β,7α-Dihydroxyroyleanone (**7**), 7α-acetoxy-6β-hydroxyroyleanone (**8**), fatty acid esters of 7α-acyloxy-6β-hydroxyroyleanone (**9**), grandidone A (**170**), and coleon U (**183**) were investigated for their effects on the proliferation of human lymphocytes induced by the phytohaemagglutinin mitogen. Except for 6β,7α-dihydroxyroyleanone (**7**), the compounds exhibited antiproliferative activity with the strongest effect observed in 7α-acetoxy-6β-hydroxyroyleanone (**8**). Comparison of the activities of 6β,7α-dihydroxyroyleanone (**7**), 7α-acetoxy-6β-hydroxyroyleanone (**8**), and fatty acid esters of 7α-acyloxy-6β-hydroxyroyleanone (**9**) showed that the character of 7α-substitution can significantly affect an antiproliferative effect. While 7α-acetoxy group was the most potent, 7α-hydroxyl signified a loss of activity. This phenomenon can be explained by the increasing lipophilicity. However, esterification of 7α-hydroxyl with fatty acids (which increased the lipophilicity of the molecule) resulted in the weaker effect explained by the size of the substituent. The authors assume that the intramolecular hydrogen bonds or even the possibility of the formation of a quinoid structure by the oxidation pathway can be responsible for the antiproliferative activity of coleon U (**183**) [49].

Coleon C (**187**) was assessed for antitumor activity on eight human tumor cell lines (human highly metastasic lung carcinoma 95-D, human melanoma A375, human cervical cancer cell line HeLa, human epidermoid carcinoma A431, human gastric cancer cell line MKN45, human hepatocellular carcinoma BEL7402, human colorectal adenocarcinoma LoVo, and human acute myeloid leukemia cell line HL-60) and two normal lines (human embryonic kidney cell line 293, and normal liver cell line L02). The results indicated that coleon C (**187**) inhibited the proliferation of human tumor cells especially HL-60, human melanoma A375, MKN45 cells, more efficiently than normal cells [120].

6-Acetylfredericone B (**57**), coleon O (**95**), and coleon G (**98**), exhibited cytotoxic effects in human multiple myeloma cancer stem cells MM-CSCs and the tumoral plasma cell line RPMI 8226. Coleon O (**95**) showed a significant activity towards both human multiple myeloma cancer stem cells and RPMI 8226 cells [12].

Ferruginol (**218**), sugiol (**220**), and 5,6-dehydrosugiol (**221**) were evaluated for their cytotoxicity towards human cervical cancer HeLa, human hepatocellular liver carcinoma HepG2, and human colon cancer HT-29. In all three cases ferruginol (**218**) was the most active, followed by 5,6-dehydrosugiol (**221**) and sugiol (**220**) [112].

Horminone (**4**), 6β,7α-dihydroxyroyleanone (**7**), 7α-acetoxy-6β-hydroxyroyleanone (**8**), coleon U-quinone (**24**), and carnosolon (**232**) were tested for their cytotoxicity against the immortalized human skin epithelial keratinocytes HaCaT to assess their antipsoriatic potential. Carnosolon (**232**) and coleon U-quinone (**24**) were the most active compounds which is consistent with the statement of González [19] that aromatic abietanes with catechol-containing molecule and carbonyl group at C7 as well as abietanes with diosphenol moiety in the ring B have the significant cytotoxic effect. Comparing the IC_50_ values of horminone (**4**), 6β,7α-dihydroxyroyleanone (**7**), and 7α-acetoxy-6β-hydroxyroyleanone (**8**) showed that the acetylated derivative 7α-acetoxy-6β-hydroxyroyleanone (**8**) displayed the strongest cytotoxic activity which corroborates with the statement of Matias et al. [53] that the lipophilicity of 7α-substituent increases the cytotoxicity of royleanone abietane diterpenes. The higher activity of horminone (**4**) compared with 6β,7α-dihydroxyroyleanone (**7**) showed that the substituent at C6 can affect the cytotoxic activity of royleanone type of diterpenes [46].

### 4.2. Antimicrobial Activity

The potential of abietane diterpenes of the genus *Plectranthus s.l.* as antimicrobial agents has been proved in several studies. 6,7-Dehydroroyleanone (**1**), 6β,7α-dihydroxyroyleanone (**7**), and 6β-hydroxy-7α-methoxyroyleanone (**12**) were investigated for antibacterial activity against *Bacillus subtilis*, and *Escherichia coli*. All of the tested compounds showed significant antibacterial activity against *E. coli* and only weak effect against *B. subtilis* [37].

6,7-Dehydroroyleanone (**1**), royleanone (**2**), 6β-hydroxyroyleanone (**3**), horminone (**4**), 6β,7α-dihydroxyroyleanone (**7**), 7α-acetoxy-6β-hydroxyroyleanone (**8**), 16-acetoxy-7α,12-dihydroxyabieta-8,12-diene-11,14-dione (**27**), 7α,12-dihydroxy-17(15→16)-*abeo*-abieta-8,12,16-triene-11,14-dione (**45**), 3β-acetoxy-6β,7α,12-trihydroxy-17(15→16),18(4→3)-*bis*(*abeo*)**-abieta-4(19),8,12,16-tetraene-11,14-dione (**55**), and coleon U (**183**) were tested for antibacterial activity against methicillin-resistant *Staphylococcus aureus* (MRSA) and vancomycin-resistant *Enterococcus faecalis* (VRE). Coleon U (**183**), 7α-acetoxy-6β-hydroxyroyleanone (**8**), and horminone (**4**) were more effective against MRSA than the positive control oxacillin. They also showed moderate activity against VRE strains. Comparison of the MIC values of the tested compounds allowed to suggest some structure–activity relationships. The presence of 12-hydroxy-*p*-benzoquinone moiety together with the oxidation at C6 or C7 seems to be essential for the antibacterial activity against MRSA and VRE. The substitution with 7α-hydroxyl present in horminone (**4**) and the simultaneous substitution with 6β-hydroxyl and 7α-acetoxyl in 7α-acetoxy-6β-hydroxyroyleanone (**8**) resulted in higher activity when compared with the 6β-hydroxylated derivative 6β-hydroxyroyleanone (**3**) or the 6β,7α-dihydroxylated derivative 6β,7α-dihydroxyroyleanone (**7**). The higher activity of 7α-acetoxy-6β-hydroxyroyleanone (**8**) is attributed to the partial lipophilicity of acetoxy group associated with the presence of the hydrophilic -COO- moiety. 6,7-Dehydroroyleanone (**1**) and royleanone (**2**) without oxygen-containing groups at the position C6 and C7 showed no activity. Comparison of the MIC values of 16-acetoxy-7α,12-dihydroxyabieta-8,12-diene-11,14-dione (**27**) with 1-acetoxyprop-2-yl side chain, 7α,12-dihydroxy-17(15→16)-*abeo*-abieta-8,12,16-triene-11,14-dione (**45**) with allyl side chain, and horminone (**4**) with isopropyl chain showed that C13 isopropyl led to the highest activity. 3β-Acetoxy-6β,7α,12-trihydroxy-17(15→16),18(4→3)-*bis*(*abeo*)**-abieta-4(19),8,12,16-tetraene-11,14-dione (**55**) exhibited weak antibacterial activity due to simultaneous substitution with hydroxyls at C6 and C7 as well as allyl side chain. Coleon U (**183**) with the most oxygenated and dehydrogenated chromophoric system spreading through the rings B and C had the highest antibacterial activity [40].

6,7-Dehydroroyleanone (**1**), royleanone (**2**), 6β-hydroxyroyleanone (**3**), 7β-hydroxyroyleanone (**14**), 7β-acetoxyroyleanone (**15**), 6β,7β-dihydroxyroyleanone (**16),** 7β-acetoxy-6β-hydroxyroyleanone (**17**), 6β,7β-dihydroxy-12-*O*-methylroyleanone (**18**), 7β-acetoxy-6β-hydroxy-12-*O*-methylroyleanone (**19**), 8α,9α-epoxy-6-deoxycoleon U-quinone (**21**), 6,12-dihydroxysapriparaquinone (**63**), 8α,9α-epoxycoleon U-quinone (**22**), coleon U-quinone (**24**), 6β-acetoxy-6α-methoxy-7-oxoroyleanone (**25**), demethylinuroyleanol (**177**), and coleon V (**204**) were tested for antibacterial activity against *E. coli*, *B. subtilis*, *Micrococcus luteus*, *Pseudomonas agarici*, and *Staphylococcus warneri*. 7β-Hydroxyroyleanone (**14**), 6β,7β-dihydroxyroyleanone (**16),** 7β-acetoxy-6β-hydroxyroyleanone (**17**), 7β-acetoxy-6β-hydroxy-12-*O*-methylroyleanone (**19**), and coleon U-quinone (**24**) showed greater zones of inhibition than the reference drug gentamycin. The weaker activity of 7β-acetoxyroyleanone (**15**) indicated that the presence of aliphatic free hydroxyl group at C6 and C7 might increase the antimicrobial activity of abietane diterpenes against the tested bacteria [41].

An antimicrobial activity against *Staphylococcus aureus*, *Vibrio cholerae*, *Candida albicans*, *Pseudomonas aeruginosa* was observed for the abietane diterpenes horminone (**4**) and 7α,12-dihydroxy-17(15→16)-*abeo*-abieta-8,12,16-triene-11,14-dione (**45**). Horminone (**4**) showed stronger antibacterial effects against *S. aureus* and *V. cholerae*, whereas 7α,12-dihydroxy-17(15→16)-*abeo*-abieta-8,12,16-triene-11,14-dione (**45**) was more effective against *C. albicans*. Both compounds showed the same effect against *P. aerigunosa* [44].

Horminone (**4**), 6β,7α-dihydroxyroyleanone (**7**), 7α-acetoxy-6β-hydroxyroyleanone (**8**), coleon U-quinone (**24**), and carnosolon (**232**) were evaluated for their antimycobacterial activity. Except for carnosolon (**232**), all of the compounds were effective against *Mycobacterium tuberculosis* [46].

6β,7β-Dihydroxyroyleanone (**16**), 7β-acetoxy-6β-hydroxyroyleanone (**17**), and coleon U-quinone (**24**) were tested for antimicrobial activity against *S. aureus*, *E. faecalis*, *E. coli*, and *P. aeruginosa*. An antimicrobial effect against Gram-positive *S. aureus* and *E. faecalis* was observed only for 7β-acetoxy-6β-hydroxyroyleanone (**17**), and coleon U-quinone (**24**). None of the test compounds had antimicrobial effects against the Gram-negative bacteria *E. coli*, and *P. aeruginosa* [54].

Coleon A (**60**), coleon A-lactone (**61**), coleon U-quinone (**24**), and coleon U (**183**) were investigated for antibacterial activity against *B. subtilis* and *Pseudomonas syringae*. While coleon A (**60**) and coleon A-lactone (**61**) were active against *B. subtilis*, coleon U-quinone (**24**) and coleon U (183) showed significant antimicrobial activity against both *B. subtilis* and *P. syringae* comparable to that of the positive control chloramphenicol [56].

The antibacterial activity of plectranthroyleanones A (**31**), B (**32**), and C (**33**) against *P. aeruginosa*, *Klebsiella pneumoniae*, *E. coli*, *B. subtilis*, and *S. aureus* was investigated in. Plectranthroyleanone A (**31**) showed weak activity against the Gram-positive bacteria *B. subtilis* and *S. aureus*, and Gram-negative bacteria *P. aeruginosa* and *K. pneumoniae*. Plectranthroyleanone B (**32**) and C (**33**) had moderate antibacterial activity against *K. pneumoniae* [58].

Scutellarioidone A (**35**), sincoetsin C (**36**), spirocoleon-7-*O*-β-D-glucoside (**107**), and 3-hydroxyspirocoleon-7-*O*-β-D-glucoside (**108**) exhibited antimicrobial activity against methicillin-resistant *S. aureus* subsp. *aureus*. Sincoetsin C (**36**) showed the strongest growth inhibitory effect. Comparison of the structures of scutellarioidone A (**35**) and sincoetsin C (**36**) indicates that methylation of hydroxyl groups at C7 and C16 increases anti-MRSA activity. The lower activity was observed in glycosides spirocoleon-7-*O*-β-D-glucoside (**107**) and 3-hydroxyspirocoleon-7-*O*-β-D-glucoside (**108**). Because of the higher anti-MRSA activity of spirocoleon-7-*O*-β-D-glucoside (**107**), the authors suggest that the hydroxylation of ring A reduces the antibacterial activity [59].

Antimicrobial activity against the Gram-positive bacteria *B. subtilis, S. aureus*, and *Streptomyces scabies* was observed in 11-hydroxy-12-oxo-7,9(11),13-abietatriene (**137**) and 7α,11-dihydroxy-12-methoxy-8,11,13-abietatriene (**228**) [86]. 

Ferruginol (**218**), sugiol (**220**), and 5,6-dehydrosugiol (**221**) exhibited antimicrobial activity against the bacteria *S. aureus*, *Streptococcus mutans*, *E. coli*, and *Salmonella typhi*, as well as the fungi *Aspergillus niger*, *Penicillium aurantiogriseum*, *C. albicans*, and *Cryptococcus neoformans*. Sugiol (**218**) was the most active compound [112].

### 4.3. Antiprotozoal Activity

7α-Acetoxy-6β-hydroxyroyleanone (**8**), 7α-formyloxy-6β-hydroxyroyleanone (**10**), and parviflorones A (**144**), C (**147**), D (**149**), E (**148**), and F (**150**) were investigated for their antiplasmodial activity against a chloroquine-resistant strain of *Plasmodium falciparum* and for an ability to inhibit β-haematin formation. The compounds were less effective than chloroquine and quinine. On the other hand, they displayed remarkable activity for the inhibition of β-haematin formation, especially 7α-formyloxy-6β-hydroxyroyleanone (**10**) and parviflorones E (**148**), and F (**150**). However, their high cytotoxicity indicates low specificity towards parasites [16].

5,6-Didehydro-7-hydroxytaxodone (**140**), taxodione (**154**), 20-deoxocarnosol (**230**), 6α,11,12-trihydroxy-7β,20-epoxy-8,11,13-abietatriene (**231**), and dehydroabietane (**237**) were tested for their antiprotozoal activity against erythrocytic schizonts of *P. falciparum*, intracellular amastigotes of *Leishmania infantum* and *Trypanosoma cruzi*, and free trypomastigotes of *Trypanosoma brucei*. 5,6-Didehydro-7-hydroxytaxodone (**140**) showed significant activity with acceptable selectivity against *P. falciparum* and *T. brucei*. Taxodione (**154**), 20-deoxocarnosol (**230**), and 6α,11,12-trihydroxy-7β,20-epoxy-8,11,13-abietatriene (**231**) exhibited non-specific antiprotozoal activity against all of the parasites tested, but they also exhibited high cytotoxicity proven on human embryonal bronchial epithelial cells MRC-5. No antiprotozoal effect was observed for dehydroabietane (**237**). The results indicated some structure–activity relationships. The antiprotozoal activity is related to oxygenated and dehydrogenated chromophoric system propagating through rings B and C. Dehydroabietane (**237**) which possesses neither oxygen-containing groups in the rings B and C nor dehydrogenation of the ring B, had no antiprotozoal effect against any of the strains tested. The moiety of extended quinones with one separatory unit as present in taxodione (**154**) is related to antiprotozoal activity, although 5,6-didehydro-7-hydroxytaxodone (**140**), 20-deoxocarnosol (**230**), 6α,11,12-trihydroxy-7β,20-epoxy-8,11,13-abietatriene (**231**) were also active. Comparison of these four compounds showed that hydroxylation at C6, C7, C11, and C12 resulted in the high antiprotozoal and cytotoxic activity. Moreover, 5,6-didehydro-7-hydroxytaxodone (**140**) with double bond between C5 and C6 and hydroxyl group at C6 (enol structure) instead of keto substitution at at C6 (keto structure) in taxodione (**154**) led to the higher selectivity [87].

7α-Acetoxy-6β-hydroxyroyleanone (**8**), 6,7-dehydroroyleanone (**1**), sugiol (**220**), coleon U (**183**), parviflorone D (**149**), (13*S*,15*S*)-6β,7α,12α,19-tetrahydroxy-13β,16-cycloabiet-8-ene-11,14-dione (**83**) were evaluated against epimastigotes of *T. cruzi*. Although parviflorone D (**149**) showed remarkable activity towards epimastigote forms of *T. cruzi*, a strong cytotoxic effect towards human lung fibroblasts LC5 and mouse fibroblasts NCTC was observed [121].

Lanugon Q (**162**) and 11,14-dihydroxy-8,11,13-abietatrien-7-one (**234**) were tested for antiprotozoal activity against *P. falciparum* and *T. brucei rhodesiense*. The cytotoxicity against rat skeletal myoblast cells L6 was determined to evaluate their selectivity towards parasites. Both compounds were active against *T. b. rhodesiense* as well as *P. falciparum*, but higher selectivity was found only towards *T. b. rhodesiense* [122].

### 4.4. Gastroprotective, Antisecretory and Antiulcer Activity

Abietane diterpenes have been found to have remarkable gastroprotective effects. 6,7-Dehydroroyleanone (**1**), royleanone (**2**), horminone (**4**), and taxoquinone (**14**) were assessed for their gastroprotective activity by oral administration using the HCl/EtOH-induced gastric lesion model in mice. All of these compounds were effective, with royleanone (**2**) showing stronger gastroprotective activity than lansoprasol [123]. 

3β-Hydroxy-3-deoxybarbatusin (**87**), and barbatusin (**89**) also had protective effects against ethanol-induced gastric lesions in mice. 3β-hydroxy-3-deoxybarbatusin (**87**) showed a greater potency than barbatusin (**89**) in lesion prevention [76]. 

Plectrinone A (**178**) was found to inhibit gastric H^+^/K^+^-ATPase. The IC_50_ value was approximately 10-fold higher than that of the classic H^+^/K^+^-ATPase inhibitor omeprazole [99]. Studies on the structure–activity relationships of flavonoids revealed that the inhibition of H^+^/K^+^-ATPase depends on the hydroxylation pattern of phenolic compounds and the number of hydroxyl groups. Protection of hydroxyls by glycosylation or methylation decreases the activity [124]. The authors assume that the presence of hydroxyls might also be crucial for the proton pump inhibitory activity of diterpenes, as plectrinone A (**178**) is a hydroquinone abietane with four free hydroxyl groups [99].

## 5. Conclusions

As illustrated in this review, abietane diterpenes have become the most studied group of secondary metabolites occurring in the genus *Plectranthus s.l.* Since the discovery of coleons A (**60**) and B (**181**) in 1963, 243 abietane diterpenes found in 91 publications have been isolated and described. Because of the large number of compounds and their remarkable structural diversity, all abietanes have been divided into nine groups. Royleanones (66 compounds), spirocoleons (70 compounds), and hydroquinones (41 compounds) belong to the most abundant groups, forming over 70% of all abietane diterpenes occurring in the genus *Plectranthus s.l.* Smaller groups include quinone methides, vinylogous quinones, 1,4-phenanthraquinones, dimeric abietanes, other phenolic abietanes, and non-phenolic abietanes.

Their wide structural diversity is linked particularly with hydroxylation and further oxidation. As shown above, these highly oxygenated compounds possess different oxygen functional groups (e.g., hydroxyls, carbonyls, carboxyls, or epoxy groups) attached at different positions of the basic abietane skeleton—except for C1. The presence of oxygen-containing groups also seems responsible for the production of rearranged abietanes, including processes such as methyl migration from the C4 to the C3 position, ring fission resulting in *seco*-abietanes, formation of derivatives with a rearranged five-membered A ring, as well as further dehydrogenation resulting in complete aromatization, which is considered the last stage before the degradation of the ring system.

## Data Availability

Not applicable.
